# Incised valleys drive distinctive oceanographic processes and biological assemblages within rhodolith beds

**DOI:** 10.1371/journal.pone.0293259

**Published:** 2023-11-13

**Authors:** Guilherme M. Castro, Rafaela P. Vargens, Lélis A. Carlos-Júnior, Fernando C. Cardoso, Paulo S. Salomon, Márcio M. B. Tenório, Alex C. Bastos, Natacha Oliveira, Renato D. Ghisolfi, Ralf T. S. Cordeiro, Rodrigo L. Moura

**Affiliations:** 1 Instituto de Biologia and SAGE/COPPE, Universidade Federal do Rio de Janeiro, Rio de Janeiro, RJ, Brazil; 2 Departamento de Biologia, Pontifícia Universidade Católica do Rio de Janeiro, Rio de Janeiro, RJ, Brazil; 3 Departamento de Oceanografia, Universidade Federal do Espírito Santo, Vitória, Brazil; 4 Departamento de Biologia, Universidade Federal Rural de Pernambuco, Recife, PE, Brazil; MARE – Marine and Environmental Sciences Centre, PORTUGAL

## Abstract

Continental shelves encompass gently sloped seascapes that are highly productive and intensively exploited for natural resources. Islands, reefs and other emergent or quasi-emergent features punctuate these shallow (<100 m) seascapes and are well known drivers of increased biomass and biodiversity, as well as predictors of fishing and other human uses. On the other hand, relict mesoscale geomorphological features that do not represent navigation hazards, such as incised valleys (IVs), remain poorly charted. Consequently, their role in biophysical processes remains poorly assessed and sampled. Incised valleys are common within rhodolith beds (RBs), the most extensive benthic habitat along the tropical and subtropical portions of the mid and outer Brazilian shelf. Here, we report on a multi-proxy assessment carried out in a tropical-subtropical transition region (~20°S) off Eastern Brazil, contrasting physicochemical and biological variables in IVs and adjacent RBs. Valleys interfere in near bottom circulation and function as conduits for water and propagules from the slope up to the mid shelf. In addition, they provide a stable and structurally complex habitat for black corals and gorgonians that usually occur in deeper water, contrasting sharply with the algae-dominated RB. Fish richness, abundance and biomass were also higher in the IVs, with small planktivores and large-bodied, commercially important species (e.g. groupers, snappers and grunts) presenting smaller abundances or being absent from RBs. Overall, IVs are unique and vulnerable habitats that sustain diverse assemblages and important ecosystem processes. As new IVs are detected by remote sensing or bathymetric surveys, they can be incorporated into regional marine management plans as conservation targets and priority sites for detailed *in situ* surveys.

## Introduction

Continental shelves cover ~9% of Earth’s surface and represent the best-known areas of the Ocean [1]. These relatively shallow areas are highly productive (19–28% of total global primary production) due to fluvio-terrigenous inputs, intense water column mixing and/or physical forcing that, in some regions, drives nutrient upwelling [1, 2]. For this reason, continental shelves are the focus of intense natural resources’ exploration within their overall gently sloped and sediment covered seascapes [2, 3]. Besides tectonic forcing, the geomorphology of continental shelves results largely from sea level fluctuations, with intense erosion and sediment transport/deposition during highstand sea levels. Conversely, they become air exposed during lowstands, when rivers cut into accumulated sediments [1, 3, 4]. Despite the centuries’ long history of exploitation and research of the coastal ocean [1–4], meso and microscale geomorphological features that do not represent navigation hazards remain poorly mapped and are poorly represented in nautical charts. For instance, in Eastern Brazil, large systems of patchy mesophotic reefs, sinkholes and other mesoscale structures remained unknown up until the 21^st^ Century [5–8].

In the outer (seaward) part of continental shelves, incised valleys (IVs) [*sensu* 9] are among such poorly known features. Recent progress in habitat mapping with acoustics and remote sensing data [e.g., 10, 11] is attracting considerable attention to such numerous and conspicuous concave structures in the Brazilian shelf [e.g., 5, 7, 10, 12–15]. Incised valleys are generally located in areas with lowered sediment deposition and often elicit biogenic carbonate precipitation, which contributes with the preservation of these structurally complex relic features [4]. The physical interference exerted by IVs can have a significant impact on water and sediment transport, ultimately enhancing both physicochemical and biological connectivity across the shelf and slope [9, 14, 16]. The complex structure of IVs also provides shelter and increased feeding opportunities for reef fishes. Furthermore, IVs may function as spawning aggregation sites for reef fish, as demonstrated by [17]. However, there remains a notable lack of comprehensive documentation regarding the biological assemblages and oceanographic processes that are driven by IVs.

While IVs are relatively sparse mesoscale structures, rhodolith beds (RB) [*sensu* 18] represent the most extensive benthic megahabitat in the outer Brazilian tropical shelf, where they cover ~167,000 km^2^ [19, 20]. Indeed, RBs represent the largest biogenic hard bottom expanses in the Southwestern Atlantic, and Eastern Brazil’s RBs are among the world’s largest [20, 21]. When compared with coral reefs, RBs are less structurally complex, but they may encompass higher diversity and biomass of several benthic and demersal groups [e.g. 21–23], including valuable fisheries resources such as lobsters and triggerfishes [8]. Rhodolith beds, which also dominate Southwestern Atlantic’s seamount tops and oceanic islands’ shelves [18, 24], provide a permeable connectivity matrix for reef-associated organisms [23]. However, the drivers of the spatial heterogeneity of RBs biological communities are still poorly assessed [21]. Quantitative biological assessments in Eastern Brazil’s vast RBs are sparse. Even for the prominent and economically significant fish assemblages associated with RBs, there is limited understanding regarding their trophic and functional structure, as well as latitudinal and cross-shelf variation patterns [23, 25, 26].

Continental shelves concentrate most human activities in the Ocean [1, 2]. Rapid economic growth is intensifying environmental impacts and inequities that exacerbate conflicts among fishers, industries (mining, oil and gas) and other users of these increasingly crowded and industrialized seascapes [27, 28]. On the other hand, Marine Spatial Planning (MSP), the public process of allocating activities to achieve ecological and socioeconomic objectives, is gaining widespread attention and holds the promise of being a practical way to organize ocean uses [27]. Besides the engagement of governmental and societal stakeholders, MSP requires biophysical information to ensure that the desired ecosystem services are indeed available and can be sustainably provided [27–29]. Habitat mapping, coupled with biodiversity and fishery assessments, represents crucial data layers for MSP. Mesoscale geomorphological features, such as the IVs examined in this study, hold promise as surrogates for identifying priority areas for conservation and management efforts.

Here we contrasted benthic and reef fish assemblages associated with IVs and the surrounding RBs in the southern part of the Espírito Santo-Abrolhos Shelf (ESA) shelf, aiming to assess: i) how their biological assemblages (benthic, fish and plankton) would differ; ii) how IVs would affect local physicochemical properties; iii) the overall significance of IVs for regional Marine Spatial Planning (MSP) and Management Plan development. We also compared our results with literature data from tropical RBs with expressive macroalgal canopies [22, 23] and recent modeling studies [25, 26].

## Methods

### Study area

**Fig 1:**
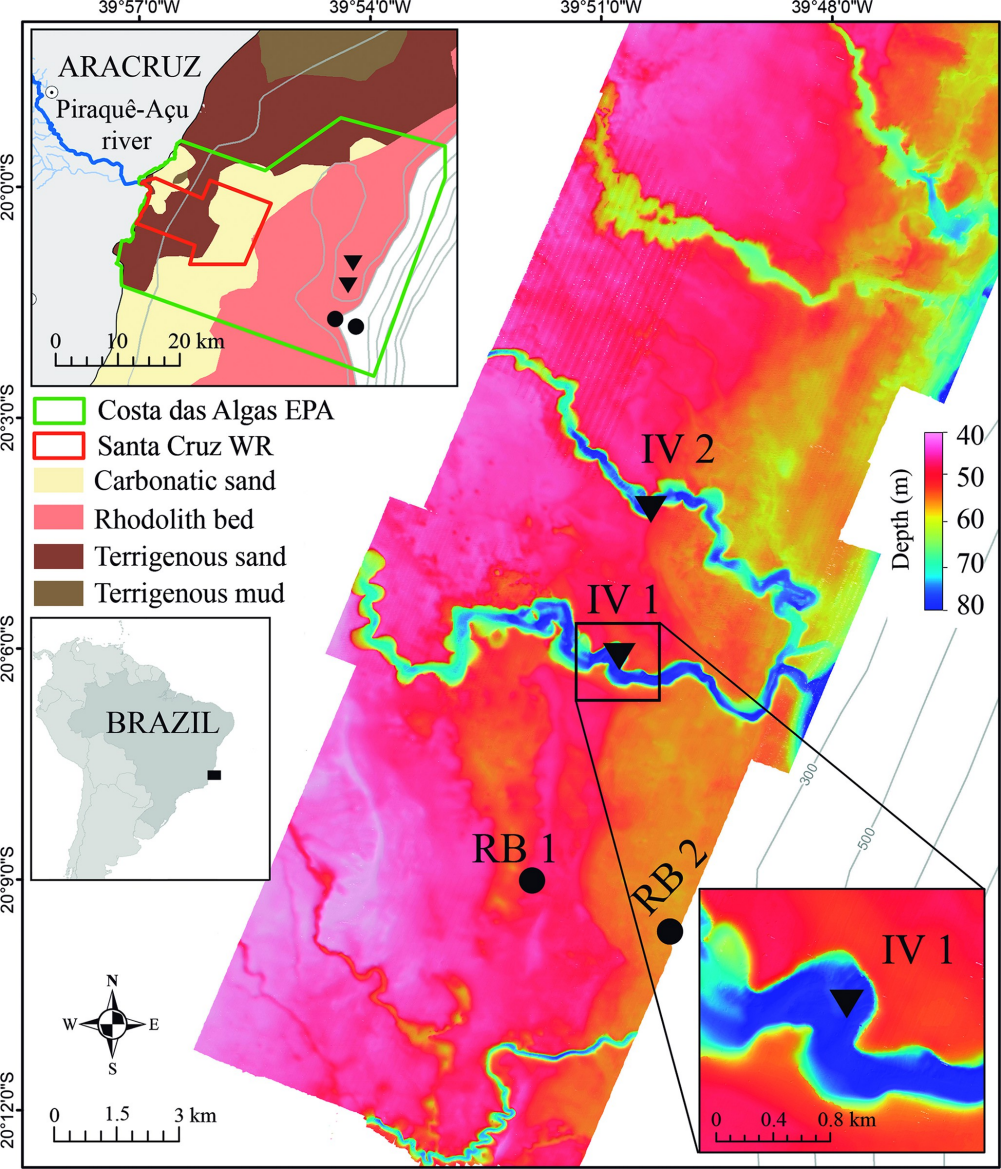
Bathymetric map of the Paleovalleys Shelf, Espírito Santo State, Brazil built with survey data from [12, 34, 35] and depicting sampling sites in incised valleys-IVs (triangles) and rhodolith beds-RB (circles). Upper left insert shows the main sedimentary facies [data from 30, 31] and Marine Protected Areas. Middle left insert shows the location of the study region (black rectangle) within Eastern Brazil. Lower right insert shows the mooring site within the incised valley (IV1). Mooring within the rhodolith bed was placed in site RB1.

The ESA shelf, located in the eastern Brazilian margin, Southwestern Atlantic Ocean, is characterized by both its highly variable shelf width (50–240 km) as well as the strong influence of the Doce River and smaller coastal drainages [7, 20]. The study region (Fig 1) comprises the Paleovalley Shelf [*sensu* 30], located in the narrow and southernmost portion of the ESA shelf, ~50 km southward to the Doce River mouth and ~35 km to the East of the Piraquê-Açu River estuary (Fig 1). The cross-shelf transition includes a shoreline with beaches, estuaries and lateritic reefs; sandy and muddy bottom in the inner-mid shelf; and rhodolith beds, low-relief reefs and incised valleys in the mid-outer shelf [30, 31].

Terrigenous sedimentation is limited in the mid-outer shelf [31] and allows for widespread carbonate sedimentation and exposition of erosive features [20, 30]. Among such relict features, IVs reach up to 600 m widths and extend along 15 km of the cross-shelf gradient, between the 30 m isobath and the shelf break at 70–80 m depth, well within the RB megahabitat (Fig 1) [12, 30, 31]. Shelf waters encompass the interface between hyposaline and turbid Coastal Water nearshore, Tropical Water in the neritic zone, and the south-flowing, warm and oligotrophic Brazil Current along the continental slope, with seasonal upwelling of colder South Atlantic Central Water [31–33]. Sampling sites are located within a multiple-use Marine Protected Area (MPA), the Costa das Algas Environmental Protection Area, and in the vicinity of a no-take MPA, the Santa Cruz Wildlife Refuge (Fig 1).

### Sampling

We sampled (April 29-May 04, 2021) the walls and channels of two IVs and two adjacent RB sites (Fig 1, Table 1) that had been previously covered by a regional survey with multibeam echosounders [12, 34, 35] (Fig 1). A mooring system (SeaGuardII^®^, Aandreaa) was deployed ~1 m above the bottom in sites RB1 and IV1 for measurements of dissolved oxygen (DO), turbidity, pH, chlorophyll a (chl-a), colored dissolved organic matter (CDOM), photosynthetically active radiation (PAR), temperature (T), density and salinity (S). A 600 kHz Acoustic Doppler Current Profiler was also moored for current measurements in vertical cells of 2 m with 10% overlap. All parameters were recorded at 10 min. intervals during 24 h. Vertical profiles were obtained with a RBR (Concerto 3) probe. Values of dissolved oxygen obtained in the moorings were corrected for temperature and salinity.

**Table 1:** Sampling sites and data collection (ADP: Acoustic Doppler Current Profiler, CTD: Conductivity, Temperature and Depth profiler, BRUV: Baited Remote Underwater Video).

	Site	Coordinates	Depth (m)	Sampling
Incised valley (IV)	IV1	20°06’06.5”S 39°50’46.0”W	77	Multiparametric/ADCP mooring, CTD casts, CCR diving (collections, photo/ video), BRUV (n = 6), water sampling.
	IV2	20°04’11.6”S 39°50’21.5”W	78	CCR diving, BRUV (n = 6).
Rhodolith bed (RB)	RB1	20°09’00.7”S 39°51’54.0”W	53	Multiparametric/ADCP mooring, CTD casts, CCR diving, BRUV (n = 5), water sampling.
	RB2	20°09’40.7”S 39°50’06.6”W	58	CCR diving, BRUV (n = 5).

Water samples from near the surface (1 m depth) and near the bottom were collected with a 5L Niskin bottle. Subsamples for flow cytometry were transferred to 2 ml cryotubes containing 34 μL of glutaraldehyde and stored in liquid nitrogen before analyses in a Cytoflex cytometer (Beckman Coulter). Autotrophs were identified and quantified following [36, 37]. The heterotrophic fraction was discriminated after staining with a fluorochrome for nucleic acids, evidencing bacterial and small eukaryote populations by size and nucleic acid levels [38], using the FlowJo software (FlowJo, LLC, OR). Richness and diversity of the pico and nano-plankton were estimated with the D_0_ (richness) and D_1_ (exponential of Shannon diversity) indices of the Hill series, following [39]. Particles between 5 and 100 μm were analyzed in an automated flow imaging system (FlowCam®, Fluid Imaging Technologies) [40] equipped with a 10X objective lens and a field-of-view 100 μm deep flow cell. Images were captured in auto-image mode with a 20 frames per second acquisition rate. Aliquots (2 L) were fixed in 2% formaldehyde and sedimented for 7 days, with subsequent removal of 90% of the supernatant. Classification and morphometric analyses were performed with the VisualSpreadSheet program. The automated imaging system records both planktonic organisms and debris (detrital organic particles). Albeit seldom reported due to difficulties in quantification with microscopy analyses, the latter can be an important source of carbon in marine food webs and thus was included in our analysis. Inverted microscopy was used to quantify the less abundant and larger phyto- and protozooplankton components. Chlorophyll pigments were obtained from 0.5–1 L seawater aliquots filtered onto 47 mm Whatman GF/F discs and stored at liquid nitrogen. Pigments were extracted by grinding the filter in 90% acetone and left in the dark at 4°C for 12-h. Samples were analyzed in spectrofluorometer (Varian, Cary Eclipse) and pigment quantification followed [41, 42].

Benthic cover (%) was estimated from orthogonal images (1 m^2^) taken at close range with a Sony RX-100 camera (4K resolution) during five diving operations (two divers and one safety officer, ~60 min. each) using closed circuit rebreathers (Megalodon, InnerSpace Systems), as well as with 10 deployments of a dropcam (0.5 m^2^) equipped with two GoPro 7 cameras (one orthogonal to the bottom and another at 45°), both equipped with light beams (BigBlue, VL10000P). Benthic images (n = 50) were annotated using 200 random points over each image with the machine-learning tool CoralNet (https://coralnet.ucsd.edu) [40]. Rhodolith density, vitality (% of living surface of each nodule) and cover by epibionts (%) were estimated from the dropcam orthogonal images following [43]. Sediment cover (%) was not considered in benthic cover estimates.

Fish assemblages were assessed with Baited Remote Underwater stereo-Videos (BRUVs) [38] using replicated diurnal (8:00–16:00) deployments (Table 1), each with 40–60 min duration (n = 22). The BRUV consists of a stainless-steel structure with two Go Pro Hero 4 cameras (1080i, 60 fps, medium FOV) inside PVC housings, both separated by 70 cm and converging at 8°. The system counts with a plastic arm (1.5 m length) that holds a bait bag with 800 g of sardines. The BRUV was calibrated using software CAL and imagery was analyzed with software EventMeasure v3.51 (SeaGIS Pty. Ltd). Relative abundances were based on the MaxN index, a conservative measure that uses the largest number of individuals of each species in a single frame [44]. Fork length of all individuals at the MaxN was measured and used to estimate biomasses through conversion constants and length/weight equations provided by FISHBASE (http://www.fishbase.org/, accessed July 2021). Total abundance and biomass were divided by the number of BRUV deployments due to unbalanced sampling effort. Species were also classified into trophic groups following [45]. Two large (>1,000 individuals) schools of sardines and small (<15 cm) jacks that were not identified were disregarded. The reef fish checklist (S1 Table) includes all species recorded with BRUVs and during rebreather dives (S1 Video).

Sampling complied with all government regulations and was carried out under a permit from Instituto Chico Mendes de Conservação da Biodiversidade (SISBIO #65055–7). Vouchers were manually collected by divers, preserved in ethanol and deposited at the cnidarian collection of Universidade Federal de Pernambuco (UFPE), Brazil. No fish was killed.

### Data analysis

Fish richness was estimated with rarefaction and extrapolation curves based on abundances, using the iNEXT package [46]. Samples were standardized based on total abundance and extrapolated to facilitate comparisons. Principal Coordinates Analysis (PCO) and Permutational Analysis of Variance (PERMANOVA) were used to explore spatial variation in assemblage structure [47, 48] and were carried out with software PRIMER v7 (Quest Research Ltd, Auckland NZ). PERMANOVA was conducted with a nested design considering the factors habitat (IV and RB) and site (IV1 and 2; RB1 and 2), this latter nested within habitat; p-value was calculated using the Monte Carlo procedure. Analyses were based on Bray–Curtis dissimilarity matrices using log-transformed (x + 1) abundance and biomass data for reef fish and benthic (%) data. A PCO based on reef fish abundance data was not shown because its configuration was similar to that based on biomass. Spearman’s rank cutoff values of 0.6 and 0.4 were used in the fish biomass and benthic cover PCOs, respectively, to show only organisms with a greater contribution to the ordination. Physicochemical data were displayed with scatterplots, boxplots and current roses, following the removal of extreme values. All raw values of depth (tides) and current speed were displayed, while additionally being smoothed using a fifth-order polynomial regression. The entire dataset used in this study is available (S1 Data).

## Results

**Fig 2:**
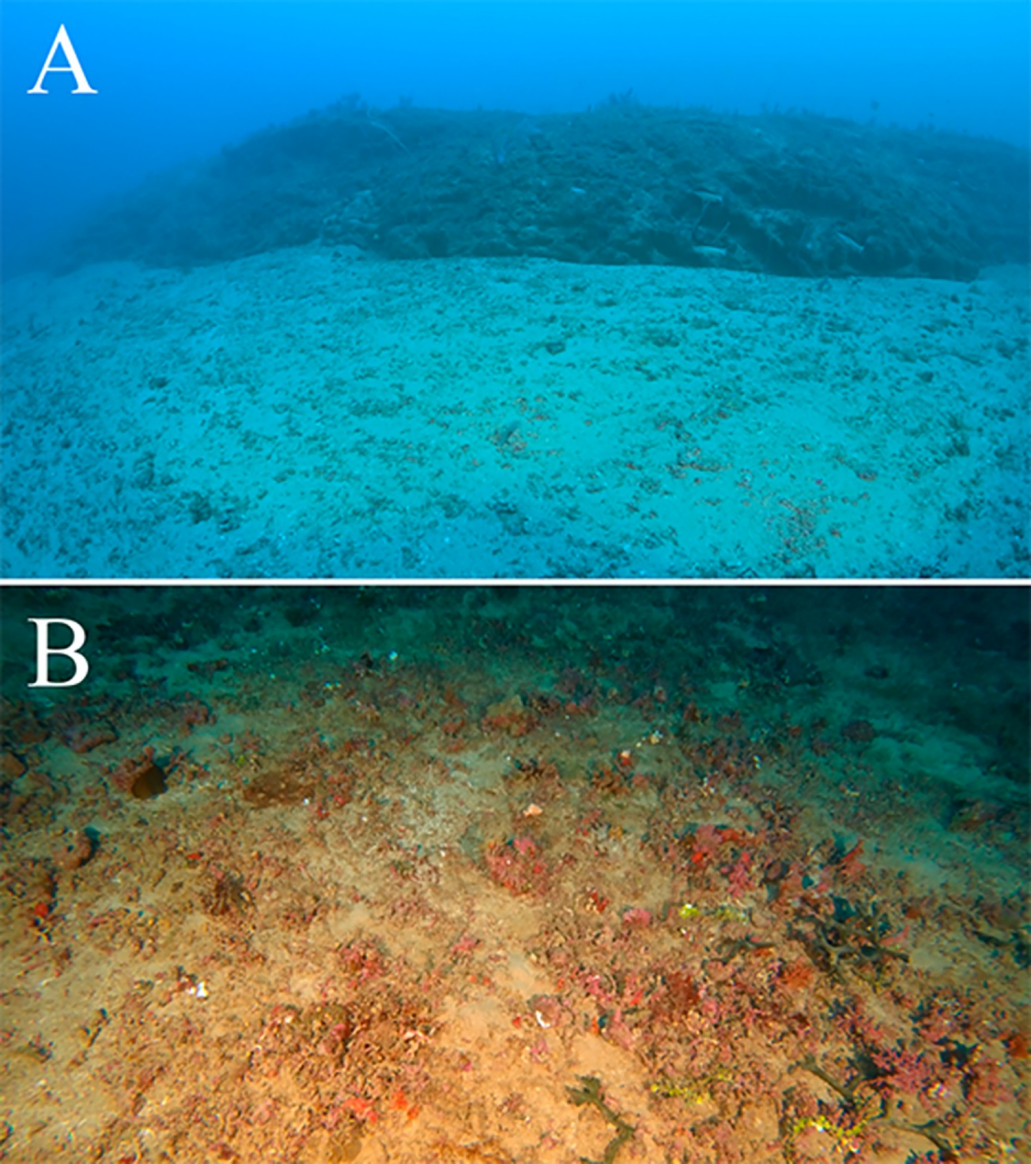
Incised valley viewed from the channel toward the wall (A, site IV1) and rhodolith bed (B, site RB1).

The two sampling sites in IVs were structurally similar, both with steep (90-45°) walls between 55–70 m depths (15–20 m height) and a complex topography with grooves, holes and crevices (Fig 2A). The valley edge was slightly convex and formed a gentle calcareous carbonate outcrop up to 1 m heigh. Outwards, the hardbottom transitioned to a flat bioclastic sand/rhodolith-dominated plain at 55–50 m depths (Fig 2B). The sandy transitional zone (IV to RB) varied between 2–10 m wide. Inwards, the wall became gradually less steep toward the flatter channel bottom (65–80 m depths), which was filled with bioclastic sands and dead/low vitality rhodoliths (Fig 2A). The two RB sites were overall flat with 30–50% of rhodolith coverage and nodules ranging between 1–5 cm diameters (max.) with overall high vitality (< 80%).

**Fig 3:**
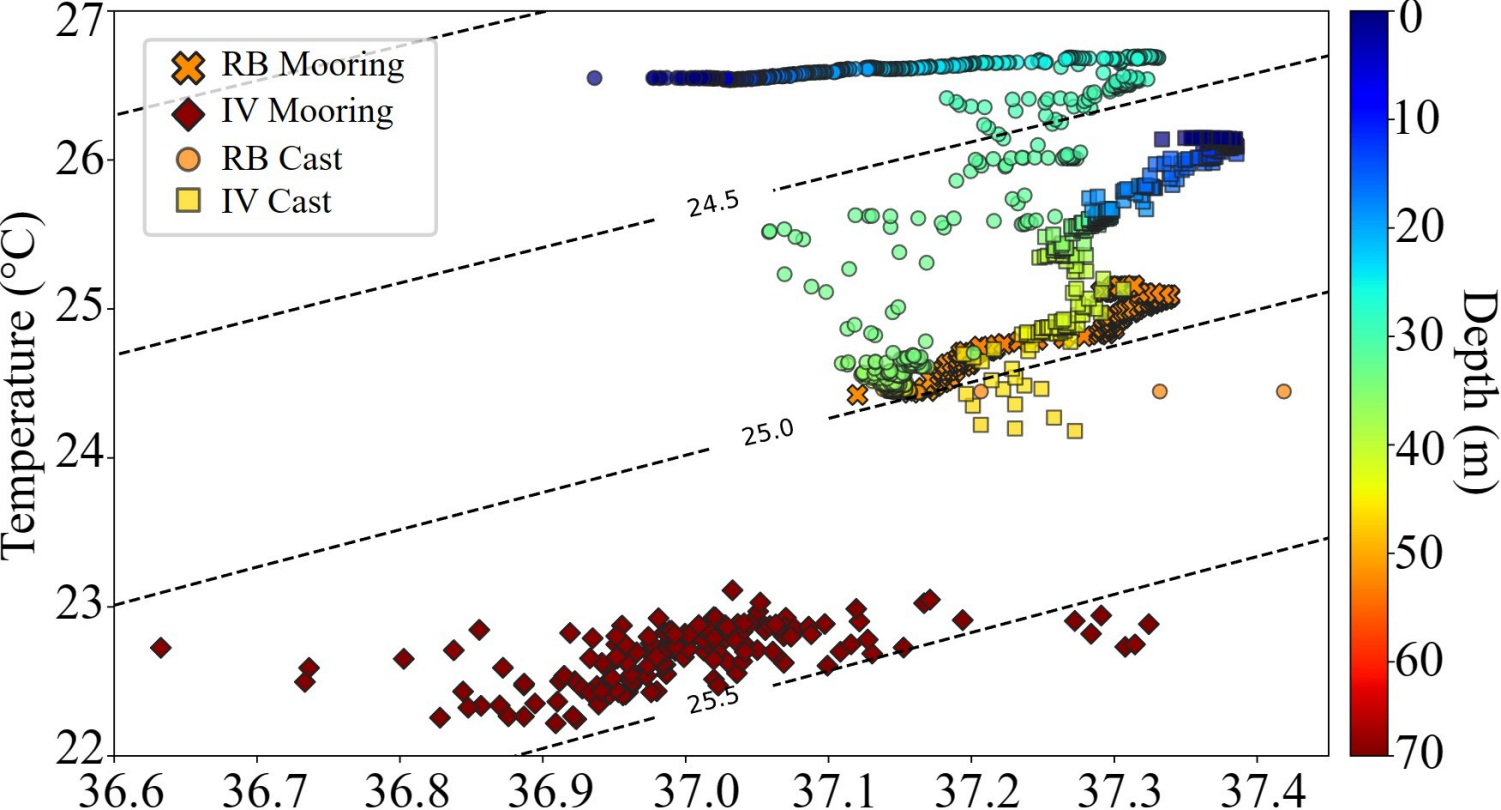
Temperature-Salinity diagram with moorings’ and casts’ data from the incised valley—IV and rhodolith bed—RB. Dashed lines represent the density contours.

**Fig 4:**
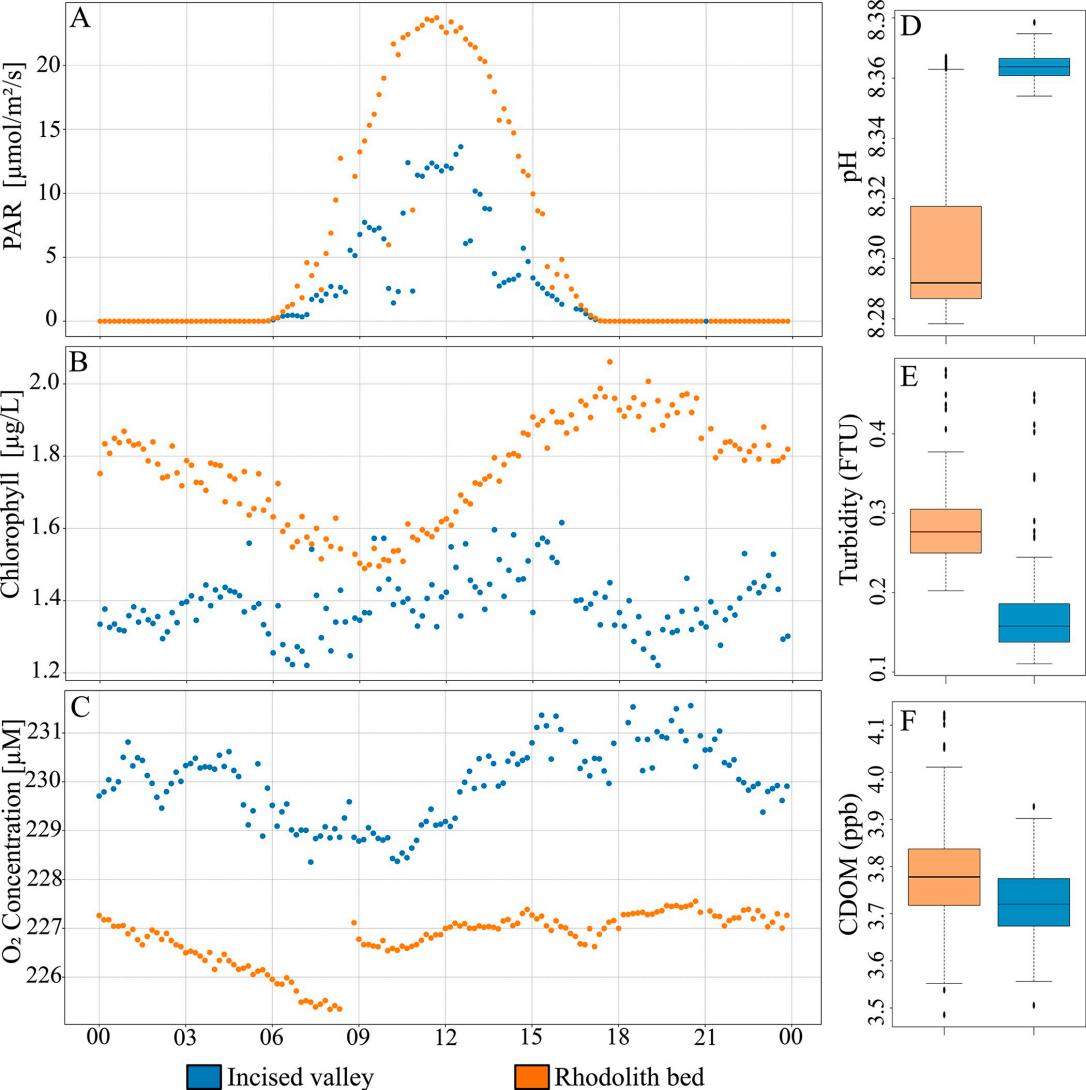
Diel and spatial variation of Photosynthetically Active Radiation—PAR (A), Chlorophyll (B) and Oxygen concentration—O_2_ (C), and spatial contrasts of pH (D), Turbidity expressed in Formazine Turbidity Units (E) and Coloured Dissolved Organic Matter—CDOM (F) in the incised valley—IV (blue) and rhodolith bed—RB (orange). The boxplot displays the median (central line), lower (Q1) and upper (Q3) quartiles within the box. Outliers are represented by isolated dots.

Water temperature and salinity data were summarized in Fig 3. Near the surface, values were similar at both sites, with temperature ranging between 26–27° C and salinity around 37, whereas near bottom water inside the IV was slightly colder (22–23°C) and less saline (< 37) than that of the adjacent RB (24–25°C, salinity >37). Overall, water temperature near the bottom was ~2°C lower than the surface in the RB and ~4°C lower than the surface in the IV. Besides discriminating most seawater parameters from inside the IV and the RB (Fig 4), moorings’ data revealed marked diel variations in photosynthetically active radiation (PAR), chlorophyll a and dissolved O_2_ concentration. Shorter daylight periods were recorded inside the IV (Fig 4A), where PAR reaching the bottom at midday corresponds to ~5% of that in the surface, contrasting with the shallower RB (~10% of surface PAR). The diel trend in Chl-a values was more evident in the RB, with lower and higher values at 9:00 and 18:00, respectively. Inside the IV, Chl-a values were consistently lower and did not show a clear diel variation (Fig 4B). Maximum and minimum DO values were respectively recorded between 18:00–20:00 and 06:00–10:00 at both sites, with higher concentrations in the IV (Fig 4C). However, daily DO means indicate an overall hypersaturation, with slightly smaller values in the warmer and more saline RB (108.2 and 105.4% daily mean, respectively). No relevant diel variation was recorded in pH, which ranged between 8.27 and 8.38, with higher and less variable values in the IV (Fig 4D). Turbidity and CDOM values also did not present diel variation and were higher in the RB (Fig 4E and 4F).

Concentrations of chlorophyll a, divinyl chlorophyll a, chlorophyll b, chlorophyll c_1_+c_2_ and phaeopigments a were higher near the surface in the IV and near the bottom in the RB (except for divinyl chlorophyll a) and tended to be higher near the bottom only in the RB (Table 2). The accessory pigments’ Chl-b/Chl-a ratio was 13 and 6 times higher near the bottom, while the Chl-c1+c2/Chl-a ratio was 4 and 2 times higher on the bottom at the IV and RB, respectively (Table 2). In the IV, *Prochlorococcus*’ divinyl chlorophyll a represented ~25% of the total Chl-a biomass near the surface and ~40% near the bottom, contrasting with RB1 (~32% and ~22%, respectively) (Table 2). Similarly, the phaeopigment-a/Chl-a ratio was 2 and 3 times higher near the bottom at the IV and RB, respectively.

**Table 2:** Pigments’ concentrations (mg/m^3^), microbial diversity and abundance (cel/L) in the incised valley (IV) and rhodolith bed (RB).

	Incised Valley		Rhodolith Bed	
	Surface	Bottom	Surface	Bottom
Chl-a	0.276	0.093	0.113	0.204
divinyl chlorophyll a	0.088	0.059	0.054	0.056
Total Chl-a (Chl-a+divinyl chlorophyll a)	0.364	0.152	0.167	0.260
% divinyl chlorophyll a (divinyl chlorophyll a*100/total Chl-a)	24.17	38.81	31.73	21.53
Chl-b	0.013	0.059	0.009	0.101
Chl-b/Chl-a ratio	0.05	0.63	0.08	0.50
Chl-c_1+_c_2_	0.010	0.015	0.008	0.026
Chl-c_1+_c_2_/Chl-a ratio	0.04	0.16	0.07	0.13
Phaeopigments-a/Chl-a ratio	0.09	0.22	0.08	0.26
D_0_ (richness)	92	222	112	280
D_1_ (exp. of Shannon diversity)	26.2	32.4	29.8	38.9
*Prochloroccocus* spp. (10⁶)	85.84	9.96	65.95	8.44
*Synechococcus* spp. (10⁶)	4.42	0.42	6.13	0.88
Eukaryotes (10⁶)	0.95	0.29	0.62	1.04
Heterotrophic bacteria (10^9^)	0.515	0.212	0.642	0.337
Autotrophic bacteria (10^9^)	0.242	0.064	0.187	0.045
Autotrophic eukaryotes (10⁶)	2.83	1.07	3.57	1.4
Protists (5–15μm) (10^3^)	24.9	61.2	28.6	52.2
Phytoplankton (>15μm) (10^3^)	4.5	2.6	7.9	9.1
Debris (10^3^)	524.3	584.9	402.9	715.4
Bacillariophyta (10^3^)	0.24	0.52	0.39	0.81
Dinoflagelatta (10^3^)	0.18	0.03	0.19	0.03
Cyanophyceae (10^3^)	0.59	0.01	0.53	0.01
Ciliophora (10^3^)	0.05	0	0.18	0.05

Counts by flow cytometry showed that *Prochlorococcus* spp. were up to 20 times more abundant than *Synechococcus* spp. at all strata (Table 2). Heterotrophic bacteria were more abundant than autotrophic ones, with similar concentrations between sites/habitats and higher abundance near the surface, akin to photoautotrophic eukaryotes. Conversely, near bottom samples were consistently more diverse (D_0_ and D_1_) (Table 2). Cell count by microscopy revealed higher concentrations of protists (5–15 μm), diatoms and debris near the bottom at both sites, with remarkably higher debris concentrations in the RB. Phytoplankton (>15 μm) concentration was associated with Chl-a, with higher values near the bottom in the RB. Other organisms such as dinoflagellates, ciliates and pico-cyanobacteria were more abundant near the surface at both habitats (Table 2).

**Fig 5:**
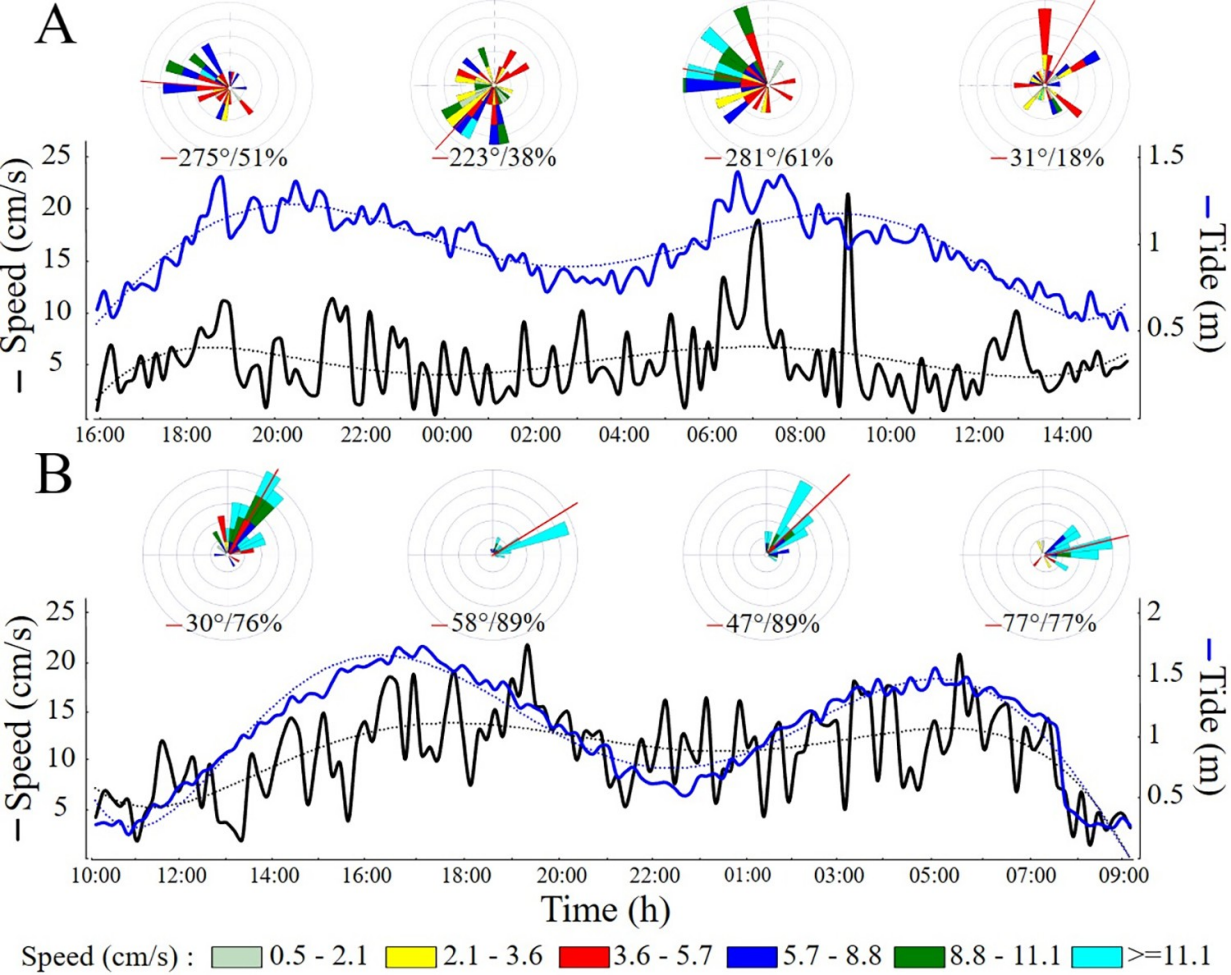
Current speed and direction near the bottom in: A) the incised valley-IV and B) the adjacent rhodolith bed-RB (sites IV1 and RB1). Current roses show current speed frequencies and directions in the ebb and flood tides; red lines represent the resultant vector. Left and right vertical axes represent horizontal speed and tidal amplitude (black and blue lines), respectively. Horizontal axes represent local time.

Current speeds were lower near the bottom, especially during ebb tides (Fig 5). Average current speed near the bottom in the RB was two times higher than inside the IV (11 and 5 cm/s, respectively). Current direction was variable inside the IV (Fig 5A), contrasting with the persistent SW flow in the RB (Fig 5B).

**Fig 6:**
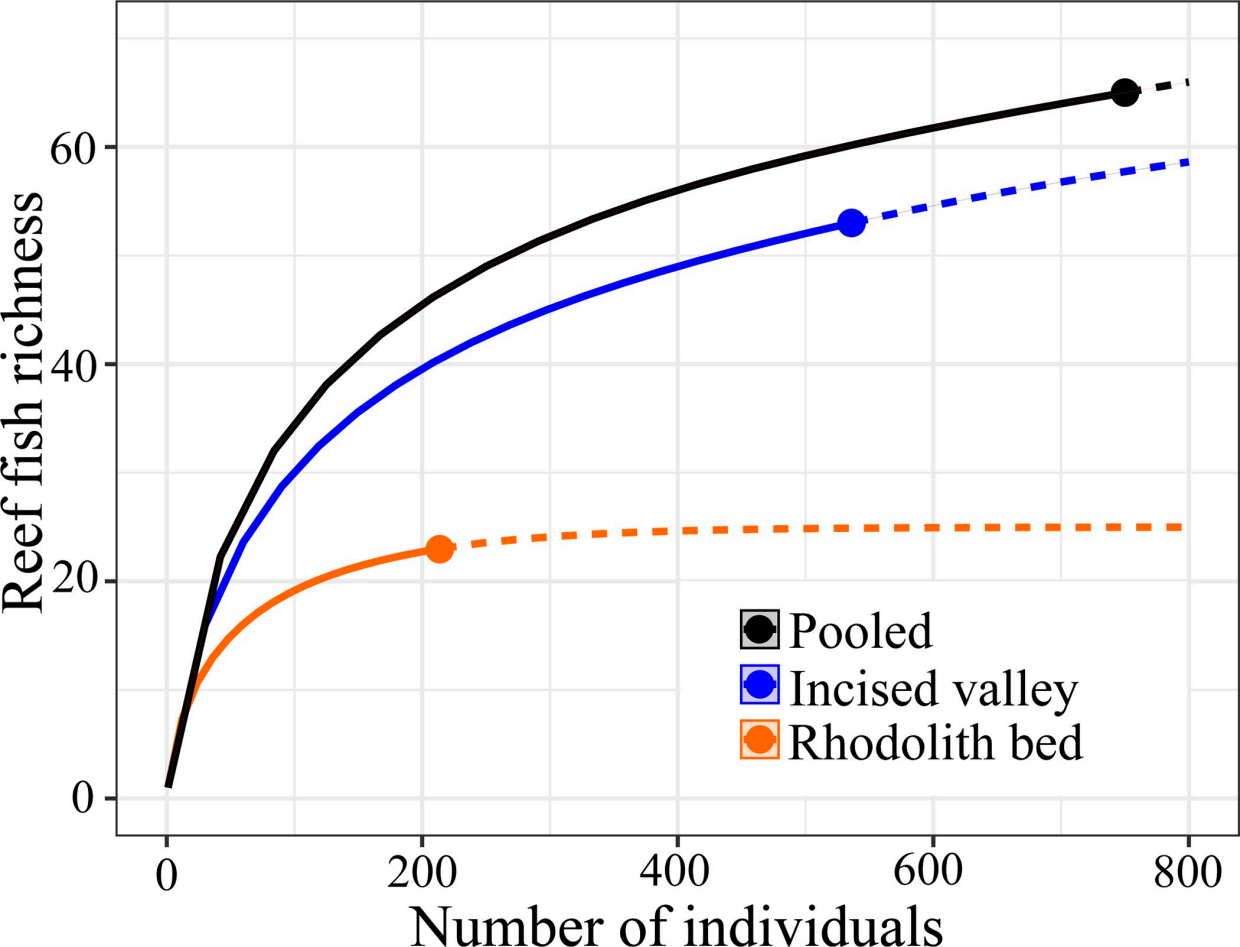
Abundance-based rarefaction (solid line) and extrapolation curves (dotted lines) for reef fishes derived from BRUV sampling in the incised valley-IV (blue) and in the rhodolith bed-RB (orange). Black curve represents a pooled estimate.

A total of 749 individual reef fishes were recorded from BRUVs’ sampling, 535 in the IV and 214 in the RB, comprising 64 species (28 families) (S1 Table), 52 (81%) in the IV and 23 (36%) in the RB (Fig 6). Most fish species were exclusive to each habitat; assemblages associated with IV and RB had 41 (84%) and 13 (57%) exclusive species, respectively. The RB assemblage included flat and/or sandy bottom specialists such as gray triggerfishes (*Balistes capriscus*), red porgies (*Pagrus pagrus*), yellowhead jawfishes (*Opistognathus aurifrons*) and sand perches (*Diplectrum formosum*). On the other hand, families Acanthuridae (surgeonfishes), Chaetodontidae (butterflyfishes), Epinephelidae (groupers), Lutjanidae (snappers), Pomacentridae (damselfishes) and Pomacanthidae (angelfishes) were only recorded in the IV.

**Fig 7:**
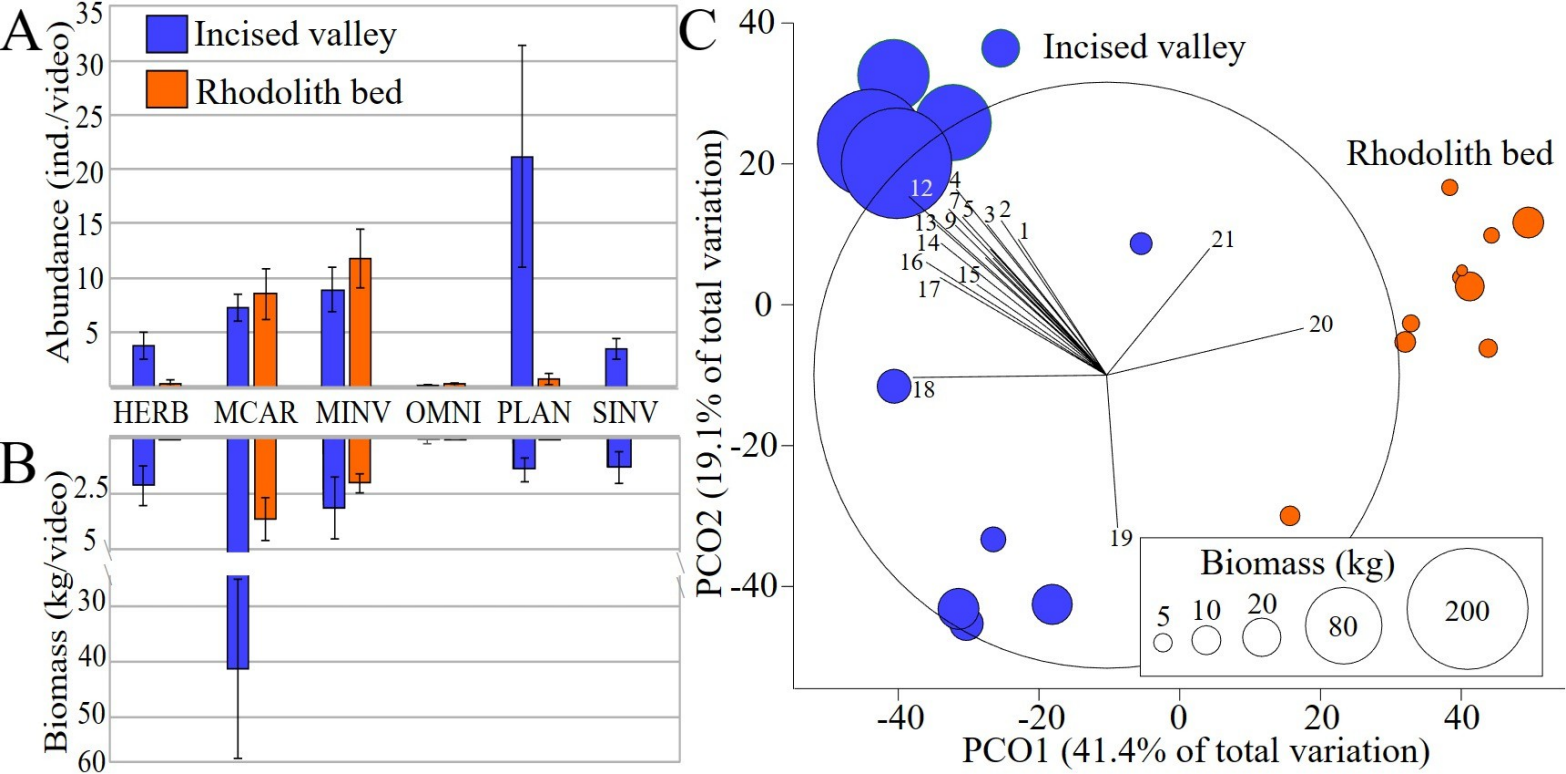
Reef fish community structure. **A and B:** Relative abundance and biomass of each reef fish trophic guild at the incised valley (IV) and rhodolith bed (RB). Error bars represent Standard Errors (SE); vertical axes with values increasing in opposite direction. Legends: HERB—Herbivores; MCAR—Macro carnivores; MINV—Mobile invertivores; OMNI—Omnivores; PLAN—Planktivores; SINV—Sessile invertivores. **C:** Principal Coordinate Analysis (PCO) with reef fish biomass data (expressed in kg). Sampling strata are color-coded in order to expose benthic habitats contrasts. Only species with the greatest contribution to the ordination are shown (1—*Chaetodon striatus*, 2—*Chromis encrysura*, 3—*Prognathodes brasiliensis*, 4—*Chaetodon sedentarius*, 5—*Mycteroperca acutirostris*, 6—*Stegastes pictus*, 7—*Acanthurus chirurgus*, 8—*Chromis flavicauda*, 9—*Bodianus pulchellu*s, 10—*Acanthurus coeruleus*, 11—*Sparisoma axillare*, 12—*Cephalopholis fulva*, 13—*Halichoeres dimidiatus*, 14—*Paranthias furcifer*, 15—*Pomacanthus arcuatus*, 16—*Sphoeroides spengleri*, 17—*Seriola dumerili*, 18—*Malacanthus plumieri*, 19—*Ptereleotris randalli*, 20—*Pagrus pagrus*, 21—*Calamus sp*.).

Relative fish abundance and biomass in the IV was two and 10 times higher than in the RB (44.6 ± 13.8 and 21.4 ± 4.0 individuals.video^-1^; 49.7 ± 16.7 and 5.6 ± 1.2 kg.video^-1^, respectively). The IV fish assemblage had a greater trophic diversity (Fig 7A) with higher abundances of planktivores such as chromis damselfishes (*Chromis jubauna*, *C*. *flavicauda* and *C*. *enchrysura*), the creole fish (*Paranthias furcifer*) and the Brazilian creole wrasse (*Clepticus brasiliensis*). The RB assemblage was dominated by macro carnivores and mobile invertivores such as porgies (*Calamus sp*. and *P*. *pagrus*), dwarf sea basses (*Serranus phoebe*, *S*. *chionaraia*, *S*. *baldwini* and *S*. *annularis*), sand perches (*Diplectrum formosum*) and goatfishes (*Pseudupeneus maculatus* and *Upeneus parvus*) (Fig 7A and 7B). Two-way nested PERMANOVAs using fish relative abundances and biomass confirmed the strong habitat effect and no site differences within habitats (IV and RB) (S2 Table).

Macro carnivores such as great amberjacks (*S*. *dumerili*), mutton snappers (*Lutjanus analis*), butterfishes (*Cephalopholis fulva*), black groupers (*Mycteroperca bonaci*) and comb groupers (*M*. *acutirostris*) accounted for the higher fish biomass in the IV (Fig 7A). Roving herbivores (~4% of total IV fish biomass) such as chubs (*Kyphosus* sp.), surgeonfishes (*Acanthurus chirurgus*, *A*. *bahianus* and *A*. *coeruleus*) and parrotfishes (*Scarus trispinosus*, *S*. *zelindae*, *Sparisoma frondosum* and *S*. *axillare*), as well as sessile invertivores (~3%) such as angelfishes (*Pomacanthus paru*, *P*. *arcuatus*, *Holacanthus tricolor* and *H*. *ciliatus*) and corallivorous butterflyfishes (*Chaetodon sedentarius*, *C*. *striatus* and *Prognathodes brasiliensis*) were largely restricted to the IV. Conversely, macro carnivores such as mackerels (*Decapterus* sp.) and mobile invertivores such as porgies and goatfishes dominated RB fish biomass (~80% of total fish biomass). Carangid macro carnivores comprised the higher biomass at both habitats, followed by the red porgy, *P*. *pagrus* in RB and the black grouper, *M*. *bonaci* in the IV.

The PCOs with fish biomass and abundance presented similar configurations, with most variation captured by the first axis (41.4 and 35% for biomass and abundance, respectively), which is related with the habitat structure (negative and positive scores related with IV and RB, respectively) (Fig 7B). The second axis captured 19.1 and 19.7% of the total variation (biomass and abundance, respectively) and seems related with the amount of sand in IV samples. All samples with negative scores in the second axis presented lower biomass and correspond to BRUV deployments near the flat and sandy bottom of the IV (Fig 7C). Conversely, positive scores were associated with its more structurally complex walls (Fig 7C).

**Fig 8:**
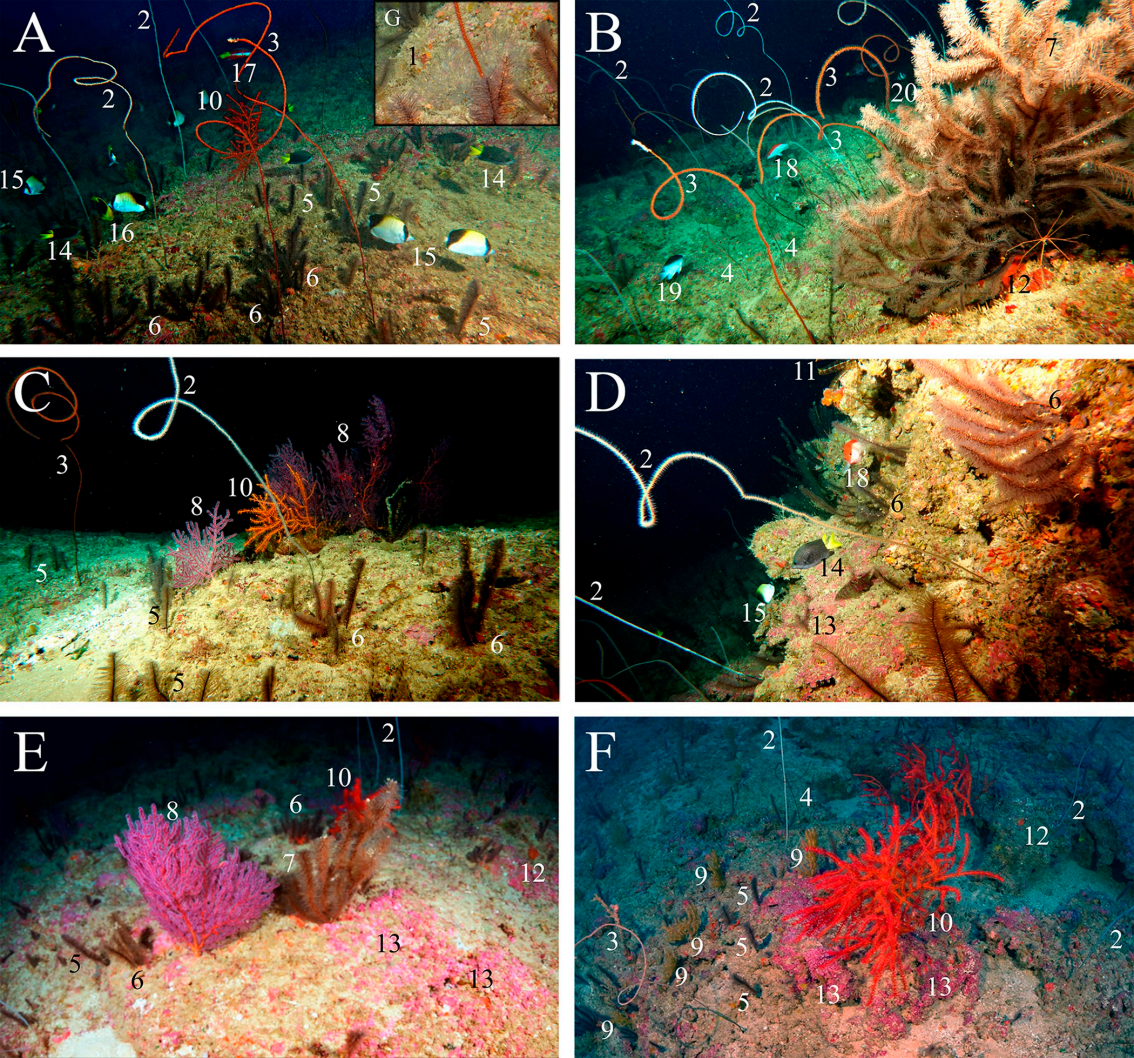
Representative species of the benthic and reef fish assemblages associated with the incised valley. Codes: 1—*Antipathus sp*., 2—*Cirripathes sp*., 3—*Stichopathes sp*.1, 4—*Stichopathes sp*. *2*, 5—*Stylopathes sp*., 6—*Tanacetipathes sp*. *1*, 7—*Tanacetipathes sp*. *2*, 8—*Muricea sp*.*1*, 9—*Muricea sp*. *2*, 10—*Swifitia sp*., 11—Crinoids, 12 –Encrusting sponges, 13—Crustose Coralline Algae (CCA), 14—*Stegastes pictus*, 15—*Chaetodon sedentarius*, 16—*Holacanthus tricolor*, 17—*Bodianus pulchellus*, 18—*Cephalopholis fulva*, 19—*Chromis enchrysura*, 20—*Chaetodon striatus*.

**Fig 9:**
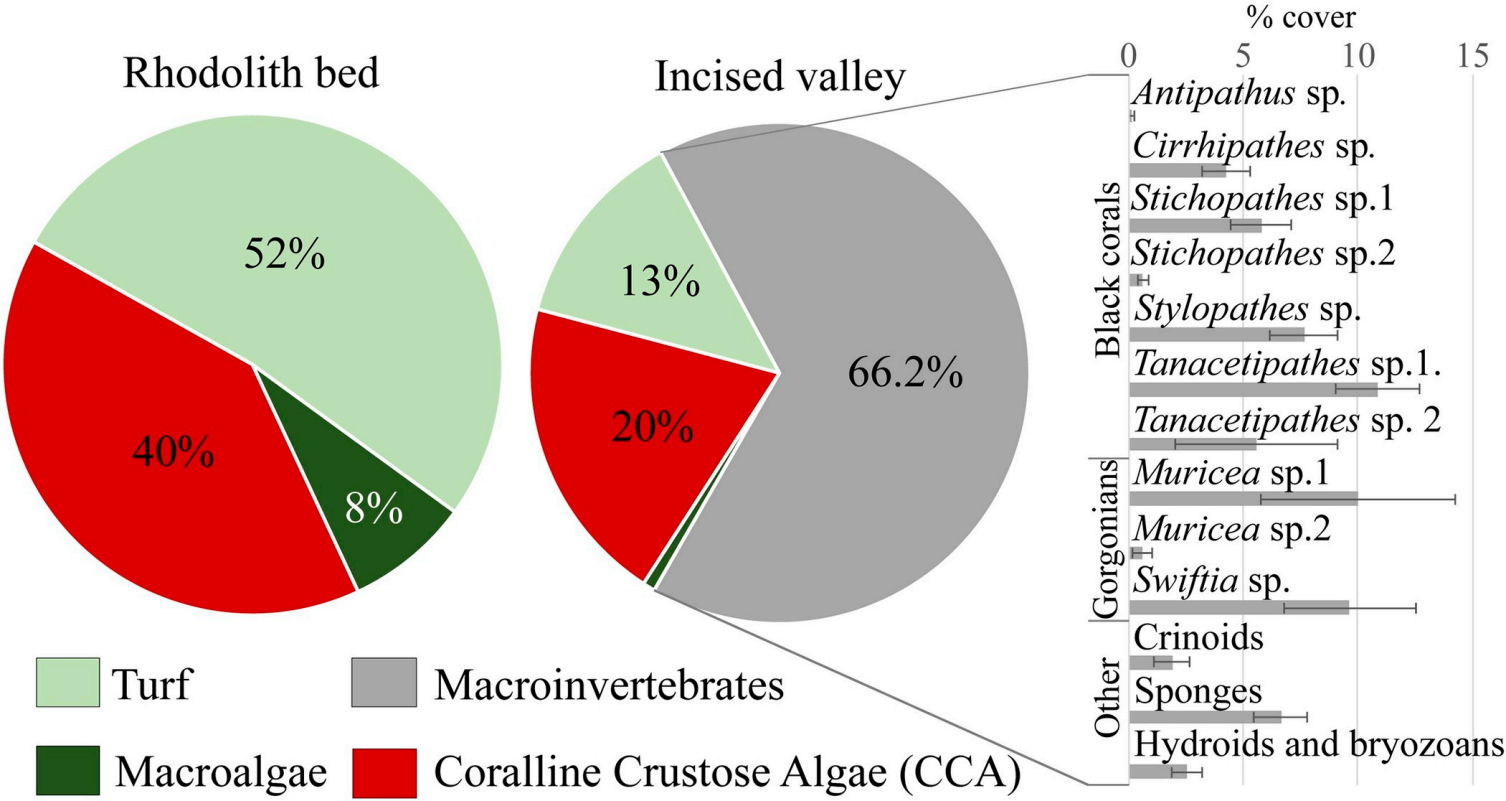
Benthic cover at the rhodolith bed (left) and incised valley (right), with a detailed account of sessile macroinvertebrates. Error bars represent Standard Errors (SE).

The benthic cover of the IV walls was dominated by black corals (order Antipatharia, 7 species from 3 families) and Plexauridae gorgonians (3 species) (Fig 8). Other macroinvertebrates such as encrusting sponges, bryozoans and crinoids covered less than 3% of the substrate each (Fig 9). Coralline Crustose Algae (CCA) covered 40 ±7.4 and 20 ±4.1% of the substrate in the RB and IV, respectively, while turf covered 52 ±18.5 and 13 ±4.2% at the RB and IV, respectively. Macroalgae were only abundant in the RB (8 ±2.6%), with less than 1% cover at the IV (Fig 9). A PCO with the benthic cover data confirms the clear discrimination between the IV and RB benthic assemblages (S2 Fig).

## Discussion

Biological productivity and biodiversity are influenced by the structural complexity of the seabed [49], but the effect of mesoscale topographic features within the vast [~167,000 km^2^; 19] and overall flat RB megahabitats off Brazil has not been previously assessed [e.g., 20, 21]. The multi-proxy survey reported herein evidenced that IVs within RBs comprise biodiversity and reef fish biomass hotspots, and also influence local oceanographic processes. Incised valleys were associated with slower water flow near the bottom, as well as with the intrusion of offshore waters with distinct planktonic assemblages. The reef fish assemblage associated with the IVs presented ten times more biomass than that of the adjacent RB, with higher abundance of small planktivores and biomass of macro carnivores targeted by fisheries. Benthic and structure-forming macroinvertebrates such as black corals and gorgonians dominated the IV and were not recorded in the turf/macroalgae-dominated RB. By contrasting our results with previous studies in RBs we also contributed to clarify the role of the ESA as a tropical-subtropical transition zone, with abrupt changes in community composition within less than two latitudinal degrees. In addition, once IV are easily detectable mesoscale features within the vast and overall flat RBs, our results demonstrate that they may be relevant targets for Marine Spatial Planning (MSP) in data-poor contexts.

### Incised valley topography and water flow

Bathymetric lows in the shelf and slope influence water circulation and sediment flow [50, 51]. While submarine canyons in the slope are major downward sediment conduits [50, 52], IV have a more limited role in cross-shelf sediment transport, which may include both up- and down-valley flows of fine sediments [e.g., 13, 51]. Sediment mobility within the Piraquê-Açu IV seems to be relatively limited, as indicated by slower near bottom currents. In addition, the IV bottom was covered by coarse carbonate sand and dead/low vitality rhodoliths, contrasting with the adjacent RB with living rhodoliths in the pebble to cobble size range [31]. The interference of the IV in near-bottom water circulation is evident from our short-term moorings and the distinctive biological assemblages inside the valley. While boundary currents usually run parallel to the coast along the shelf-break, near-bottom currents in valleys and canyons cut across these flows and are usually aligned to their topography [e.g., 16, 50, 52]. Current speed may be either reduced or increased by valleys, depending on depth and tidal range, meandering patterns, and continental slope orientation relative to the boundary current. For instance, in Brazil’s Equatorial Margin, which is dominated by macrotides and the west-flowing North Brazil Current, two large (1–8 and 1–5 km wide) IVs in shallow water (>30 m depth) drive transversal (N-S) tide-induced near-bottom currents with 25 cm/s mean speed, i.e. nearly half of the average current speed over the shelf [5, 13, 14]. The Piraquê-Açu IV flow was also tidally influenced and reduced water speed, but induced penetration of deeper and colder water into the shelf, contrasting with the Equatorial Margin valleys. The RB adjacent to the Piraquê-Açu valley is dominated by the warm and saline Tropical Water typical of the Eastern Brazilian shelf, with minor or no influence from Coastal Waters with strong terrigenous signatures [32]. Inside the IV, our Temperature-Salinity data reveals a mix with the colder and less saline South Atlantic Central Water, which ranges between 5–20°C and 34.6–36.2 salinity [32]. Also, water flow inside the IV followed its orientation, which is overall transversal to the southward energetic flow of the Brazil Current that dominates the outer shelf and upper slope [11]. Internal waves and extreme weather events may drive water flow and sediment transport along valleys [34, 51], but their influence within the Piraquê-Açu IV and other IVs is unknown and deserve further attention.

### Plankton and pigments

While submarine canyons often pump nutrients into the euphotic zone and affect microbial, zooplankton and micronekton assemblages [50], the influence of IVs over these assemblages has not been previously assessed. *Prochlorococcus* spp., the smallest photosynthetic organisms, dominate oligotrophic waters and their relatively high abundance is a reliable signature of ocean waters’ intrusion into the shelf [53]. As expected, the concentration of these cyanobacteria was ~8 times higher on the surface than near the bottom, with lower Chl-b/Chl-a and Chl-c/Chl-a ratios at the surface, at both sites. Near bottom concentrations of *Prochlorococcus* were only slightly higher inside the IV than in the RB, but divinyl chlorophyll a from *Prochlorococcus* represented ~40% of the total chlorophyll a concentration near the bottom in the former, suggesting that photoacclimation contributes to such higher pigment concentrations (e.g. Chl-b and chl-c). Although *Prochlorococcus* can synthesize some chlorophyll b [53], increased ratios of accessory pigment-to-chlorophyll a seem to be more related to autotrophic eukaryotes with higher relative proportions of Chl-c_1_+c_2_ (chromophytes) and Chl-b (chlorophytes). In addition, elevated concentrations of Chl-a and accessory chlorophylls near the bottom, especially in the RB, are likely related with higher abundances of diatoms and other larger phytoplankton. Turbidity and CDOM values, as well as phaeopigments-a’ concentrations were also relatively higher near the bottom in the RB and may be associated with organic matter degradation and higher macroalgal debris concentrations [23]. The higher abundance of small protists and *Prochlorococcus* spp. inside the Piraquê-Açu IV adds to the idea that it conducts offshore water from the upper slope into the shelf.

### Fish and benthic assemblages

Structurally complex habitats provide a greater variety of microhabitats for sessile and demersal biota, and generally encompass more biodiversity and biomass than sediment covered habitats [49, 54]. Despite being relatively flat, tropical RBs are a major reef fish habitat, especially for micro/macro invertivorous fish that feed amongst the calcareous nodules, as well as for carnivores/planktivores that feed in the water column [23]. Due to restraints of conventional diving below 30 m depths, remote video is the standard quantitative approach for fish sampling in RBs [44, 55]. However, the sampling coverage within the >30° latitudinal degrees where RBs occur off Brazil was restricted to two surveys in the tropical Northeastern continental margin [23, 56], with seven and 137 BRUV deployments each, and one survey covering two areas within the subtropical southeastern coast [25], with a total of 100 deployments. Therefore, generalizations and comparisons must be done cautiously, as remarked by [23]. For instance, while [56] reported a species poor fish assemblage near Todos os Santos Bay (northern Bahia), [23] showed that fish richness in the Abrolhos Bank RBs exceed that recorded in the region’s coral reefs due to higher turnover along the larger and heterogeneously covered (macroalgae, turf, CCA, invertebrates) RB realm.

Here, we showed that IVs are biomass and diversity hotspots for fishes within RBs in the ESA Shelf, with two- and ten-times higher fish abundance and biomass in the IV than in the adjacent RB. The sandy flat in the valley bottom and near its crest provides suitable habitat for psammophylous species (e.g. *Ptereleotris randalli*) and contributed to the “valley effect” of enhanced diversity. Planktivores are a minor component of RB fish assemblages, where their role as a major pelagic-reef linkage [e.g. 57] seems to be limited [23]. However, the IV assemblage was numerically dominated by small-bodied diurnal planktivores (*Chromi*s spp.). This trend seems related to offshore water pumping coupled with the valley’s structural complexity and may drive the relatively high biomass of macro carnivores such as jacks, groupers and snappers (Carangidae, Serranidae and Lutjanidae, respectively), which feed on small planktivores. Herbivores comprised a minor component of the RB and IV assemblages, which was dominated by a few large roving fish (e.g., *Acanthurus* spp., *Sparisoma* spp.). Such lowered abundance of herbivores in RBs has also been noted in the Abrolhos Bank and may be associated with the dominance of unpalatable macroalgae in this habitat [22, 23]. Macro carnivores and mobile invertivores were more abundant in the RB, but their biomass was higher in the IV. This reflects the preponderance of small serranids and medium-sized porgies and goatfishes (Sparidae and Mullidae, respectively) in the RB, while carnivores that eat larger prey were more abundant in the valley (e.g., jacks, groupers and snappers). The fish assemblage structure in both RB and IV indicates that most energy flowing through fishes in these habitats comes either from small benthic prey or from secondary production in the water column, with a limited contribution from benthic herbivory, contrasting with shallow water reefs [23, 54].

A typical mesophotic benthic assemblage [58] with high macroinvertebrate cover was recorded in IV walls, contrasting with the adjacent RB dominated by turf and macroalgae. The relatively high abundance of black corals and gorgonians (65% of substrate cover) seems largely related with substrate stability, once RB are mobile [18] and therefore less suitable for long-lived, slow-growing and large/structurally complex sessile macroinvertebrates. Shading from walls and channeling of colder offshore water with food and propagules may also contribute to the increased diversity and abundance of black corals and gorgonians in the IV [46]. Remarkably, other IVs so far studied in Brazil’s Equatorial Margin [e.g., 13, 59] did not present such heterogeneous benthic and fish assemblages relative to their surroundings. The level of biophysical connectivity with the continental slope and the surrounding benthic habitats (reefs or RBs) may explain such differences, but further investigation is needed to interpret geographic variation in valley-effects.

### Tropical-subtropical transition

Biological assemblages associated with Southwestern Atlantic RBs may vary considerably along their wide latitudinal and depth range, from shallower high-energy to deeper low-energy settings [18, 19]. Light at bottom, which depends on depth and turbidity, as well as CaCO_3_ saturation state and water temperature, are among the main predictors of RB occurrence and community structure variation [e.g., 19, 23, 60]. The ESA shelf is in a tropical-subtropical transition [25] with sharp differences in sedimentation patterns [61], sea surface temperature and water column stratification (S1 Fig) within less than two latitudinal degrees. For instance, among the region’s reefs, the dominant coral in the northerly Parcel dos Abrolhos reefs, *Mussismillia braziliensis*, does not occur in the Esquecidos reefs (70 km south of the former) and southwards. Conversely, fish species that are abundant in subtropical reefs such as the dusky grouper (*Epinephelus marginatus*) do not occur, or are extremely rare, northwards of Espírito Santo [45]. Rhodolith beds’ size and composition also present a sharp contrast within the ESA shelf [61]. Macroalgae cover in RBs is at least 5 times higher eastward to the Abrolhos reefs than in the southerly Paleovalleys Shelf [21, 23, 31], where macroalgae diversity, richness and cover is concentrated in the nearshore lateritic reefs.

The sharp transitions within the ESA shelf are also evident in fish assemblage structure, once several species are consistently more abundant in either its north or southward portion (S1 Fig). For instance, the red porgy (*P*. *pagrus*), a commercially important macro carnivore in Southeastern Brazil, was the most abundant fish in the RB adjacent to the Piraquê-Açu IV and in BRUV surveys in Southeastern Brazil RBs [25], but it was not recorded in any of the 13 RB sites sampled by [23] in the northern portion of the ESA Shelf (n = 90 BRUV deployments). Northwards, the red porgy was also absent from comprehensive assessments in the tropical shelves of Rio Grande do Norte, Paraiba, Pernambuco, Alagoas and Bahia states [56, 62]. However, recent macroecological assessments based on niche modeling underrated the significance of the tropical-subtropical transition within the ESA shelf [25, 26]. For instance, [25] wrongly predicted that the red porgy occurs throughout the Brazilian shelf and that this fish and the “Brazilian rhodolith bed megahabitat are ecologically interlinked and interdependent”. Predictions of severe niche erosion at lower latitudes due to global warming are also almost certainly to be invalid, once the red porgy does not occur (or is extremely rare) along most of Eastern Brazil’s tropical RB realm. Further consideration of environmental data that is not available from large-scale databases of climate predictors [e.g. 63] is needed for a more comprehensive understanding of distribution and abundance patterns in RB’s biological assemblages, especially because survey data is lacking or is spatially biased. As remarked by [60], the absence of standardized sampling data provides weak frameworks to further discuss macroecological drivers. Potentially important predictors for the presence and abundance of species associated with RBs may include habitat structure (nodules density, size, shape and composition), sediment type and proportion, macroalgal canopy, among others. From our results, fish richness is twice higher in the northern RBs off the Abrolhos reefs (Bahia state) than in the Paleovalleys Shelf (Espírito Santo state) (S1 Fig). This contrasts with the idea that biogeographic patterns of fishes associated with RBs are similar to those of coral/rocky reefs [26], once the maximum reef fish richness recorded in Espírito Santo rocky reefs decreases sharply both north and southwards [45]. We also did not find evidence for higher richness of herbivorous fish in subtropical RBs [26].

### Marine Spatial Planning (MSP) and protected areas

In Brazil, MSP is being gradually incorporated in the country’s legal and policy framework [27, 28]. Nevertheless, the legal declaration of relatively isolated marine protected areas (MPAs), mostly established after *ad hoc* consultation (e.g., no-take National Parks and Biological Reserves) or from stakeholder claims (e.g., multiple-use Extractive Reserves), still represent the most significant (yet clearly insufficient) marine management tools [e.g. 7, 27, 28]. Here, we showed that IVs are biodiversity and biomass hotspots within the dominant hard bottom megahabitat of the Brazilian shelf, and can serve as good surrogates for areas with increased biodiversity and/or related with processes that sustain ecological and evolutionary persistence. Since IVs may be detected from remote sensing and acoustic surveys [11, 34], both of which achievable in relatively short timeframes, these and other mesoscale geomorphological features [e.g. 6, 7, 10, 12] should be incorporated in MSP as priority sites for *in situ* surveys.

Zoning schemes of existing MPAs may also benefit from the detection of mesoscale features even when biodiversity data is weak or unavailable. The present study, carried out in the multiple-use Costa das Algas Environmental Protection Area (1,149 km^2^), illustrates this perspective. Declared in 2010 and aiming at the management of fisheries and other natural resources’ uses, the MPA still lacks a fishers’ register and a management plan with specific regulations [64]. Fisheries are artisanal, with shrimp trawling concentrated in nearshore soft bottom [65, 66]. In the mid and outer shelf, where bioclastic sands and rhodolith beds predominate [65, 31], fisheries target pelagic resources (e.g. *Scomberomorus*, *Coryphaena*) with encircling nets and surface/mid-water longlines, as well as reef-associated groupers (Serranidae), snappers (Lutjanidae) and grunts (Haemulidae) captured with longlines set near the bottom [65, 66]. The IVs are important fishing spots (locally known as “regos”, “fendas” or “canais”) that have been used by locals for several decades, and their full closure to fisheries would be disruptive and unrealistic. However, IVs occupy a small fraction of the RB habitat within the MPA, and at least some of the valleys [31] could be reliably be set as permanent or temporary no-take zones, once fishers recognize their importance as “nurseries” [66]. Alternative or complementary measures could include a ban of fishing and anchoring gear that may damage the IV benthic assemblages, as well as winter bans of groupers’ fishing, once valleys are well-known spawning sites, and the reproductive seasons of large serranids are well known [17, 66].

## Conclusions

Recent progresses in remote sensing and habitat mapping are revealing that incised valleys formed during lowstand sealevel are common mesoscale features along the tropical Brazilian shelf and elsewhere. Our multi-proxy survey in the so-called Paleovalleys shelf (Espírito Santo, Brazil) indicates that these features increase cross-shelf connectivity, biodiversity and fish biomass in the overall flat rhodolith beds that dominate the mid and outer tropical and subtropical Brazilian shelf. The studied valleys presented lowered current speeds near the bottom and seem to function as conduits that bring water and propagules from the upper slope up to the mid shelf. The stable and structurally complex valley walls comprise a benthic assemblage dominated by black corals and gorgonians that usually occur in deeper water. This rich benthic assemblage type was so far unknown from the Brazilian shelf, and contrasts sharply with the turf-macroalgae covered seascape typical of rhodolith beds. Fish richness and biomass were highly increased in the valleys. Numerous small planktivores (e.g. chromis damselfishes) and high biomass of large carnivores (groupers and snappers) characterize valley’s fish assemblages, which are structurally and functionally distinct from those of the adjacent rhodolith beds dominated by pelagic carnivores (jacks) and benthic invertivores (porgies). Comparisons with data from the northern part of the Abrolhos Bank highlighted the significance of the studied region as a tropical-subtropical transition, and calls the attention for the need for more data about the variation in the structure of rhodolith bed communities before assessing their potential environmental drivers and projecting environmental changes’ scenarios. Incised valleys may be significant biophysical proxies for biodiversity in data-poor marine spatial planning and may be specifically targeted by the Management Plan of the Costa das Algas Environmental Protection Area.

## Supporting information

S1 Fig Contrasts between the Northern (N) and Southern (S) Rhodolith Beds (RB) within the tropical-subtropical transition of the Espírito-Santo Abrolhos (ESA) Shelf. A: Temperature-Salinity plots showing stronger summer stratification in the South and a more mixed water column during the winter; B: Summer and winter Sea Surface Temperatures (dashed lines represents the 100m isobath); C: Rarefaction (solid) and extrapolation curves (dotted) of reef fish richness in the Abrolhos Bank (N) (data from [22]) shown in orange and in the Paleovalley Shelf (S) shown in blue; D: Video frames showing latitudinal contrasts in algal canopies and fish assemblages.
(DOCX)

S2 Fig Principal Coordinate Analysis (PCO) with benthic cover data (%). Sampling strata are color-coded in order to expose benthic habitats contrasts. Only species with the greatest contribution to the ordination are shown.
(DOCX)

S1 Table Checklist of fishes recorded during the survey in the Piraquê-Açu incised valley and adjacent rhodolith bed. (DOCX)

S2 Table Full results of the Permutational Analysis of Variance (PERMANOVA) contrasting reef fish biomass and abundance in the incised valley and adjacent rhodolith bed. (DOCX)

S1 Video Supplementary video file. (MP4)

S1 Data Raw data. (ZIP)
