# Supplementary material for: Incised valleys drive distinctive oceanographic processes and biological assemblages within rhodolith beds
Source: PLoS One. 2023 Nov 13;18(11):e0293259. doi: 10.1371/journal.pone.0293259 (PMC10642839; doi:10.1371/journal.pone.0293259)
Supplement: S1 Fig — A: Temperature-Salinity plots showing stronger summer stratification in the South and a more mixed water column during the winter; B: Summer and winter Sea Surface Temperatures (dashed lines represents the 100m isobath); C: Rarefaction (solid) and extrapolation curves (dotted) of reef fish richness in the Abrolhos Bank (N) (data from [22]) shown in orange and in the Paleovalley Shelf (S) shown in blue; D: Video frames showing latitudinal contrasts in algal canopies and fish assemblages. (DOCX) [file pone.0293259.s001.docx]

**
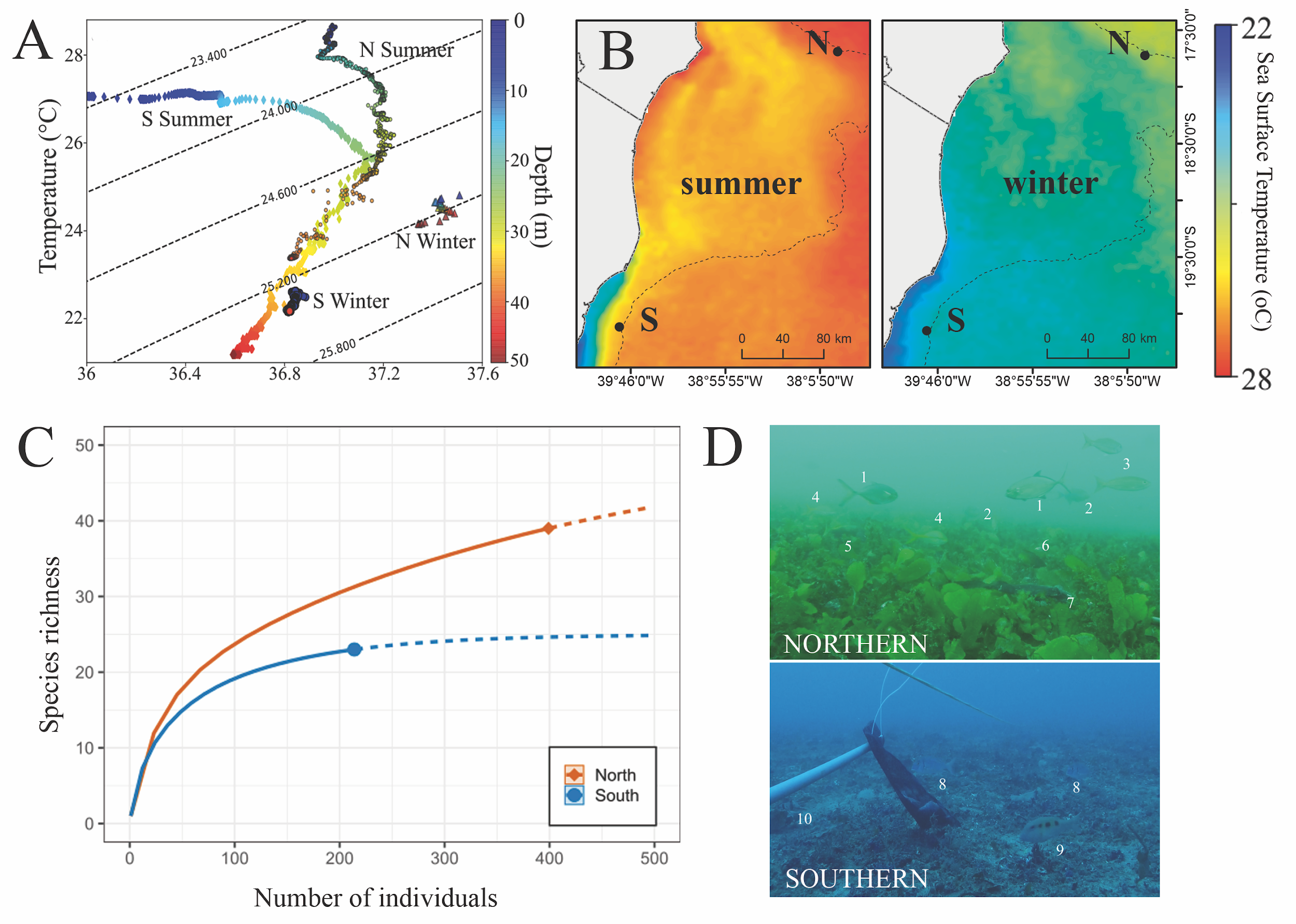
**

S1 Fig. Contrasts between the Northern (N) and Southern (S) Rhodolith Beds (RB) within the tropical-subtropical transition of the Espírito-Santo Abrolhos (ESA) Shelf. A: Temperature-Salinity plots showing stronger summer stratification in the South and a more mixed water column during the winter; B: Summer and winter Sea Surface Temperatures (dashed lines represents the 100 m isobath); C: Rarefaction (solid) and extrapolation curves (dotted) of reef fish richness in the Abrolhos Bank (N) (data from [22]) shown in orange and in the Paleovalley Shelf (S) shown in blue; D: Video frames showing latitudinal contrasts in algal canopies and fish assemblages. Fish identifications: 1 - *Caranx crysos*, 2 - *Balistes vetula*, 3 - *Rhomboplites aurorubens*, 4 - *Ocyurus chrysurus*, 5 - *Haemulon plumierii*, 6 - *Cephalopholis fulva*, 7 - *Gymnothorax moringa*, 8 - *Pagrus pagrus*, 9 - *Pseudupeneus maculatus*, 10 - *Serranus phoebe*.
