# Supplementary material for: Incised valleys drive distinctive oceanographic processes and biological assemblages within rhodolith beds
Source: PLoS One. 2023 Nov 13;18(11):e0293259. doi: 10.1371/journal.pone.0293259 (PMC10642839; doi:10.1371/journal.pone.0293259)
Supplement: S2 Fig — Sampling strata are color-coded in order to expose benthic habitats contrasts. Only species with the greatest contribution to the ordination are shown. (DOCX) [file pone.0293259.s002.docx]

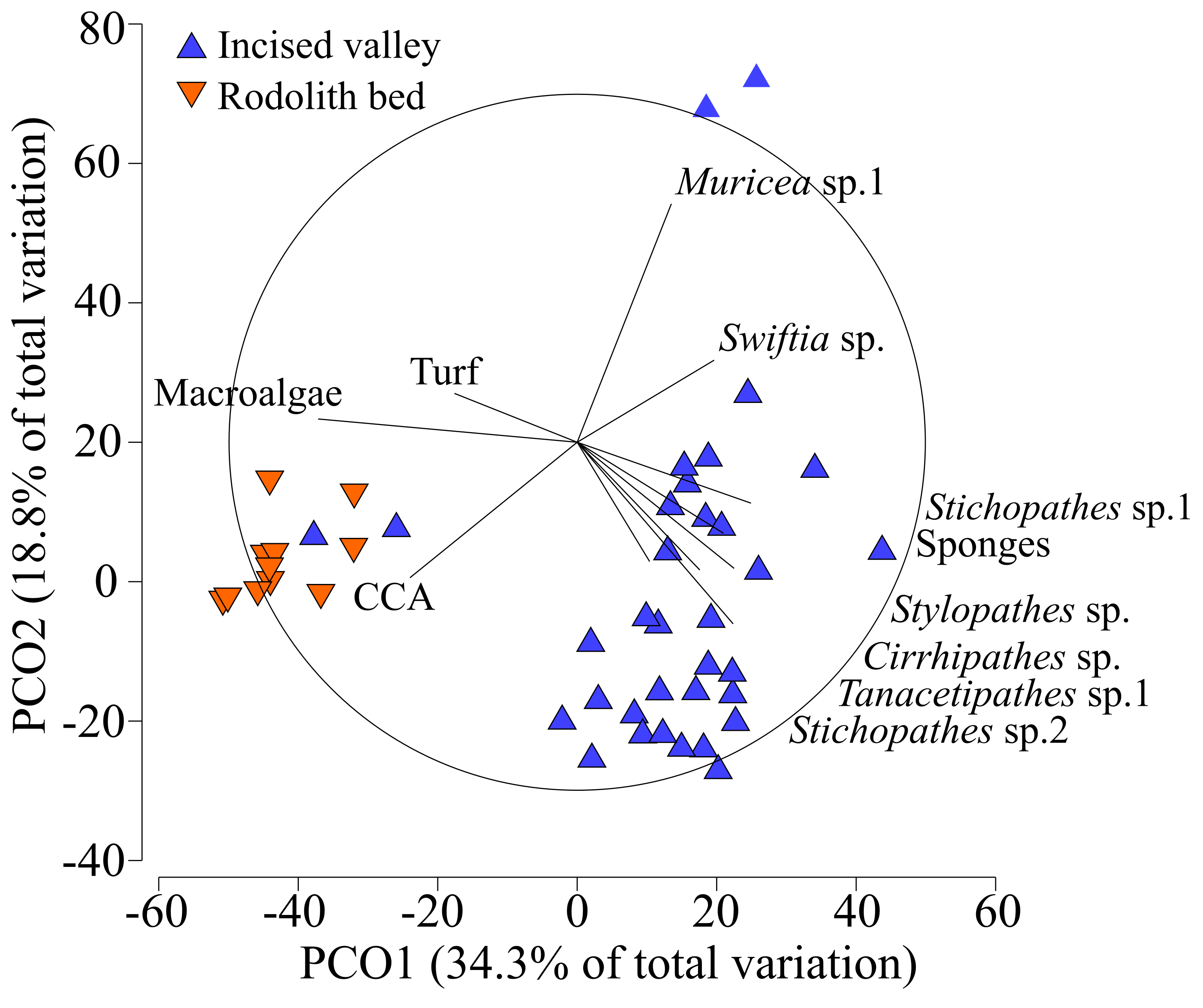


S2 Fig. Principal Coordinate Analysis (PCO) with benthic cover data (%). Sampling strata are color-coded in order to expose benthic habitats contrasts. Only species with the greatest contribution to the ordination are shown.
