# Supplementary material for: Incised valleys drive distinctive oceanographic processes and biological assemblages within rhodolith beds
Source: PLoS One. 2023 Nov 13;18(11):e0293259. doi: 10.1371/journal.pone.0293259 (PMC10642839; doi:10.1371/journal.pone.0293259)
Supplement: S1 Table — (DOCX) [file pone.0293259.s003.docx]

S1 Table. Checklist of fishes recorded during the survey in the Piraquê-Açu incised valley and adjacent rhodolith bed.

|  |  | | | | | **Rhodolith bed** | | | **Incised valley** | |  |
| --- | --- | --- | --- | --- | --- | --- | --- | --- | --- | --- | --- |
|  | Distribution | Max size (cm) Sampled/ Literature | | Max. Depth (m) | Trophic Guild | | MaxN ± SE | Biomass ± SE | MaxN ± SE | Biomass ± SE | |
|  |  |  |  |  |  |  |  |  |  |  |  |
| **Holocentridae** |  |  |  |  |  | |  |  |  |  | |
| *Holocentrus adscensionis* | WA, OIB, MAR, EA | 26/35 | | 200 | MIN | | NR | | 1.9 ± 1.2 | 0.4 ± 0.3 | |
| *Sargocentron bullisi* |  |  |  |  |  | |  |  |  |  | |
| **Apogonidae** |  |  |  |  |  | |  |  |  |  | |
| *Apogon americanus* | BR, OIB | 7/12 | | 63 | PLA | | NR | | VR | | |
| *Phaeoptyx* *pigmentaria* | WA, OIB, EA | 5/8 | | 70 | PLA | | NR | | VR | | |
| [**Gobiidae**](https://en.wikipedia.org/wiki/Gobiidae) |  |  | |  |  | |  |  |  |  | |
| *Ptereleotris randalli* | BR | 10/12 | | 60 | PLA | | 0.4 ± 0.3 | * | 0.3 ± 0.2 | * | |
| **Pomacentridae** |  |  | |  |  | |  |  |  |  | |
| *Chromis enchrysura* | BR, OIB | 20/13.5 | | 125 | PLA | | NR | | 2.6 ± 1.3 | 0.1 ± 0.1 | |
| *Chromis flavicauda* | BR, OIB | 10/7 | | 120 | PLA | | NR | | 6.3 ± 3.9 | 0.1 ± 0.1 | |
| *Chromis jubauna* | BR, OIB | 14/7 | | 71 | PLA | | NR | | 8.7 ± 5.9 | 0.3 ± 0.2 | |
| *Stegastes pictus* | BR, WA, OIB | 10/7.5 | | 85 | HER | | NR | | 0.8 ± 0.4 | * | |
| **Opistognathidae** |  |  | |  |  | |  |  |  |  | |
| *Opistognathus aurifrons* | BR, OIB | 5/14 | | 65 | PLA | | 0.3 ± 0.3 | * | NR | | |
| **Echeneidae** |  |  | |  |  | |  |  |  |  | |
| *Echeneis naucrates* | CT | 70/110 | | 50 | MCA | | NR | | VR | | |
| **Carangidae** |  |  | |  |  | |  |  |  |  | |
| *Carangoides bartholomaei* | WA, OIB, EA | 32/90 | | 70 | MCA | | 0.1 ± 0.1 | 0.1 ± 0.1 | NR | | |
| *Caranx crysos* | WA, OIB, MAR, EA | 41/70 | | 100 | MCA | | 1.3 ± 0.6 | 0.6 ± 0.2 | 0.1 ± 0.1 | 0.1 ± 0.1 | |
| S1 Table (continued). Checklist of fishes recorded during the survey in the Piraquê-Açu incised valley and adjacent rhodolith bed. | | | | | | | | | | | |
|  |  | | | | | **Rhodolith bed** | | | **Incised valley** | |  |
|  | Distribution | Max size (cm) Sampled/ Literature | | Max. Depth (m) | Trophic Guild | | MaxN ± SE | Biomass ± SE | MaxN ± SE | Biomass ± SE | |
|  |  |  |  |  |  |  |  |  |  |  |  |

| *Caranx latus* | WA, OIB, MAR, EA | 58/100 | 140 | MCA | | NR | | 0.1 ± 0.1 | 0.3 ± 0.3 | |
| --- | --- | --- | --- | --- | --- | --- | --- | --- | --- | --- |
| *Decapterus sp.* | WA, MAR, EA | 36/21 | 90 | MCA | | 5.4 ± 1.9 | 2.4 ± 0.8 | NR | | |
| *Seriola dumerili* | CT | 140/150 | 360 | MCA | | 0.1 ± 0.1 | * | 1.8 ± 0.6 | 33.3 ± 13.5 | |
| **Sphyraenidae** |  |  |  |  | |  |  |  |  | |
| *Sphyraena guachancho* | WA, OIB, EA | 54/200 | 100 | MCA | | 0.2 ± 0.2 | 0.2 ± 0.2 | NR | | |
| **Bothidae** |  |  |  |  | |  |  |  |  | |
| *Bothus* sp. | WA | 25.5/16 | 121 | MIN | | 0.2 ± 0.1 | * | 0.1 ± 0.1 | * | |
| [**Fistulariidae**](https://en.wikipedia.org/wiki/Cornetfish) |  |  |  |  | |  |  |  |  | |
| *Fistularia* sp. | CT | 136/180 | 200 | MCA | | 0.2 ± 0.1 | 0.2 ± 0.2 | 0.2 ± 0.1 | 0.1 ± 0.1 | |
| [**Scombridae**](https://pt.wikipedia.org/wiki/Scombridae) |  |  |  |  | |  |  |  |  | |
| *Scomberomorus* sp. | WA, OIB | 87/184 | 140 | MCA | | NR | | 0.1 ± 0.1 | 0.4 ± 0.4 | |
| **Labridae** |  |  |  |  | |  |  |  |  | |
| *Bodianus pulchellus* | WA, OIB, EA | 25/38 | 120 | MIN | | NR | | 1.1 ± 0.4 | 0.1 ± 0.1 | |
| *Clepticus brasiliensis* | BR, OIB | 25/30 | 62 | PLA | | NR | | 0.1 ± 0.1 | * | |
| *Cryptotomus roseus* | WA, OIB, MAR | 5/13 | 66 | HER | | 0.3 ± 0.3 | * | NR | | |
| *Halichoeres dimidiatus* | BR, OIB | 27/20 | 71 | MIN | | NR | | 0.7 ± 0.2 | 0.1 ± 0.1 | |
| *Halichoeres sazimai* | BR | 17/23 | 190 | MIN | | 0.1 ± 0.1 | * | 1.1 ± 0.8 | * | |
| *Scarus trispinosus* | BR | 59/35.5 | 30 | HER | | NR | | 0.1 ± 0.1 | 0.5 ± 0.5 | |
| *Scarus zelindae* | BR, OIB | 37/33.2 | 55 | HER | | NR | | 0.1 ± 0.1 | 0.1 ± 0.1 | |
| *Sparisoma axillare* | BR, OIB | 33/37 | 45 | HER | | NR | | 0.5 ± 0.3 | 0.2 ± 0.1 | |
| *Sparisoma frondosum* | BR, OIB | 33/34.5 | 45 | HER | | NR | | 0.3 ± 0.2 | 0.3 ± 0.2 | |
| S1 Table (continued). Checklist of fishes recorded during the survey in the Piraquê-Açu incised valley and adjacent rhodolith bed. | | | | | | | | | | |
|  |  | | | | **Rhodolith bed** | | | **Incised valley** | |  |
|  | Distribution | Max size (cm) Sampled/ Literature | Max. Depth (m) | Trophic Guild | | MaxN ± SE | Biomass ± SE | MaxN ± SE | Biomass ± SE | |
|  |  |  |  |  |  |  |  |  |  |  |

| **Mullidae** |  |  |  |  | |  |  |  |  | |
| --- | --- | --- | --- | --- | --- | --- | --- | --- | --- | --- |
| *Pseudupeneus maculatus* | WA, OIB | 27/30 | 90 | MIN | | 2.6 ± 1.4 | 0.5 ± 0.2 | 0.9 ± 0.8 | 0.1 ± 0.1 | |
| *Upeneus parvus* | WA | 22/30 | 112 | MIN | | 2.8 ± 2.8 | 0.2 ± 0.2 | NR | | |
| **Kyphosidae** |  |  |  |  | |  |  |  |  | |
| *Kyphosus* sp. | WA, OIB, MAR, EA | 54/70 | 55 | HER | | NR | | 0.1 ± 0.1 | 0.3 ± 0.3 | |
| **Epinephelidae** |  |  |  |  | |  |  |  |  | |
| *Cephalopholis fulva* | WA, OIB | 34/30 | 218 | MCA | | NR | | 1.9 ± 0.5 | 0.5 ± 0.2 | |
| *Epinephelus morio* | WA | 36/125 | 300 | MCA | | NR | | 0.1 ± 0.1 | 0.1 ± 0.1 | |
| *Mycteroperca acutirostris* | WA | 56/98 | 110 | MCA | | NR | | 0.3 ± 0.1 | 0.7 ± 0.3 | |
| *Mycteroperca bonaci* | WA, OIB | 106/122 | 70 | MCA | | NR | | 0.3 ± 0.2 | 3.5 ± 2.0 | |
| *Paranthias furcifer* | WA, OIB, MAR, EA | 32/40 | 70 | PLA | | NR | | 3.3 ± 1.3 | 0.9 ± 0.4 | |
| **Serranidae** |  |  |  |  | |  |  |  |  | |
| *Diplectrum formosum* | WA, OIB | 27/30 | 80 | MCA | | 1.1 ± 0.4 | 0.1 ± 0.1 | 0.2 ± 0.2 | 0.1 ± 0.1 | |
| *Serranus annularis* | WA, OIB | 8/9 | 70 | MIN | | 0.5 ± 0.3 | * | 0.3 ± 0.2 | * | |
| *Serranus baldwini* | WA, OIB | 6/12 | 80 | MIN | | 0.5 ± 0.3 | * | NR | | |
| *Serranus chionaraia* | WA | 5/5 | 90 | MIN | | 0.6 ± 0.4 | * | NR | | |
| *Serranus phoebe* | WA, OIB | 16/20 | 400 | MIN | | 0.8 ± 0.4 | * | 1.3 ± 0.2 | * | |
| **Chaetodonidae** |  |  |  |  | |  |  |  |  | |
| *Chaetodon sedentarius* | WA, EA | 15/15 | 92 | SIN | | NR | | 1.6 ± 0.5 | 0.1 ± 0.1 | |
| *Chaetodon striatus* | WA, OIB | 13/16 | 65 | SIN | | NR | | 0.6 ± 0.2 | * | |
| S1 Table (continued). Checklist of fishes recorded during the survey in the Piraquê-Açu incised valley and adjacent rhodolith bed. | | | | | | | | | | |
|  |  | | | | **Rhodolith bed** | | | **Incised valley** | |  |
|  | Distribution | Max size (cm) Sampled/ Literature | Max. Depth (m) | Trophic Guild | | MaxN ± SE | Biomass ± SE | MaxN ± SE | Biomass ± SE | |
|  |  |  |  |  |  |  |  |  |  |  |

| *Prognathodes brasiliensis* | BR, OIB | 11/7.5 | 65 | SIN | NR | | 0.4 ± 0.2 | * |
| --- | --- | --- | --- | --- | --- | --- | --- | --- |
| **Pomacanthidae** |  |  |  |  |  |  |  |  |
| *Centropyge aurantonotus* | BR, WA, OIB, EA | 6.5/7.5 | 200 | HER | NR | | 0.2 ± 0.2 | * |
| *Holacanthus ciliaris* | WA, OIB | 30/45 | 120 | SIN | NR | | 0.2 ± 0.1 | 0.1 ± 0.1 |
| *Holacanthus tricolor* | WA, OIB | 9.5/35 | 92 | SIN | NR | | 0.2 ± 0.1 | * |
| *Pomacanthus arcuatus* | WA, OIB | 46/60 | 30 | SIN | NR | | 0.4 ± 0.2 | 1.0 ± 0.5 |
| *Pomacanthus paru* | WA, OIB, MAR | 40/41 | 100 | SIN | NR | | 0.1 ± 0.1 | 0.2 ± 0.2 |
| **Malacanthidae** |  |  |  |  |  |  |  |  |
| *Malacanthus plumieri* | WA, OIB, MAR | 43/70 | 153 | MCA | NR | | 1.5 ± 0.3 | 0.5 ± 0.1 |
| **Haemulidae** |  |  |  |  |  |  |  |  |
| *Anisotremus surinamensis* | WA, OIB | 52/60 | 60 | MIN | NR | | 0.2 ± 0.1 | 0.4 ± 0.3 |
| **Lutjanidae** |  |  |  |  |  |  |  |  |
| *Lutjanus analis* | WA | 73/94 | 95 | MCA | NR | | 0.3 ± 0.2 | 1.6 ± 1.0 |
| *Lutjanus jocu* | WA, OIB, MAR | 45/128 | 70 | MCA | NR | | 0.2 ± 0.2 | 0.3 ± 0.3 |
| *Ocyurus chrysurus* | WA, OIB, EA | 47/86 | 180 | MCA | NR | | 0.1 ± 0.1 | 0.1 ± 0.1 |
| [**Ephippidae**](https://pt.wikipedia.org/wiki/Ephippidae) |  |  |  |  |  |  |  |  |
| *Chaetodipterus faber* | WA | 44/91 | 35 | MIN | NR | | 0.5 ± 0.5 | 1.3 ± 1.3 |
| **Sciaenidae** |  |  |  |  |  |  |  |  |
| *Pareques acuminatus* | WA | 17/23 | 54 | MIN | NR | | VR | |

| S1 Table (continued). Checklist of fishes recorded during the survey in the Piraquê-Açu incised valley and adjacent rhodolith bed. | | | | | | | | | | |
| --- | --- | --- | --- | --- | --- | --- | --- | --- | --- | --- |
|  |  | | | | **Rhodolith bed** | | | **Incised valley** | |  |
|  | Distribution | Max size (cm) Sampled/ Literature | Max. Depth (m) | Trophic Guild | | MaxN ± SE | Biomass ± SE | MaxN ± SE | Biomass ± SE | |
|  |  |  |  |  |  |  |  |  |  |  |
| **Acanthuridae** |  |  |  |  | |  |  |  |  | |
| *Acanthurus bahianus* | BR, WA, OIB, MAR | 24/30 | 71 | HER | | NR | | 0.4 ± 0.3 | 0.1 ± 0.1 | |
| *Acanthurus chirurgus* | WA, OIB, MAR, EA | 36.5/35 | 70 | HER | | NR | | 0.8 ± 0.3 | 0.2 ± 0.1 | |
| *Acanthurus coeruleus* | WA, OIB, MAR | 35/36 | 71 | HER | | NR | | 0.4 ± 0.2 | 0.3 ± 0.2 | |
| **Sparidae** |  |  |  |  | |  |  |  |  | |
| *Calamus* sp. | WA | 35/38 | 86 | MIN | | 0.7 ± 0.2 | 0.2 ± 0.1 | 0.3 ± 0.2 | 0.3 ± 0.1 | |
| *Pagrus pagrus* | WA, EA, OIB | 29/91 | 250 | MIN | | 2.5 ± 0.8 | 0.5 ± 0.2 | NR | | |
| [**Ogcocephalidae**](https://pt.wikipedia.org/wiki/Ogcocephalidae) |  |  |  |  | |  |  |  |  | |
| *Ogcocephalus vespertilio* | WA | 18/30 | 200 | MIN | | NR | | 0.1 ± 0.1 | * | |
| **Ostraciidae** |  |  |  |  | |  |  |  |  | |
| *Acanthostracion sp.* | WA, OIB, EA | 47/55 | 80 | OMN | | NR | | 0.1 ± 0.1 | 0.1 ± 0.1 | |
| **Balistidae** |  |  |  |  | |  |  |  |  | |
| *Balistes capriscus* | WA, OIB, EA | 43/35 | 100 | MIN | | 0.4 ± 0.2 | 0.5 ± 0.2 | NR | | |
| *Balistes vetula* | WA, OIB, MAR, EA | 53.5/50 | 111 | MIN | | NR | | 0.1 ± 0.1 | 0.3 ± 0.3 | |
| **Monacanthidae** |  |  |  |  | |  |  |  |  | |
| *Stephanolepis hispidus* | WA, OIB, EA | 14/27.5 | 293 | OMN | | 0.2 ± 0.1 | * | NR | | |
| **Tetraodontidae** |  |  |  |  | |  |  |  |  | |
| *Lagocephalus laevigatus* | WA, EA | 32/100 | 180 | MCA | | 0.1 ± 0.1 | 0.1 ± 0.1 | NR | | |
| *Sphoeroides camila* | SWA, OIB | 10/30 | 70 | MIN | | NR | | 0.6 ± 0.2 | * | |

**NR** – Not recorded; **VR** – Visual Record; * - < 0.05

Distribution: **WA** - Western Atlantic, **MAR** - Mid Atlantic Ridge, **OIB** - Oceanic Islands of Brazil, **BR** - Brazilian Coast Endemics**, SA** - Southwestern Atlantic, **EA** – Eastern Atlantic; **CT** - Circumtropical

Trophic Guild: **HER –** Herbivores, **MCA –** Macro carnivores, **MIN** – Mobile invertebrate feeders, **OMN** – Omnivore, **PLA** – Planktivore, **SIN** – Sessile invertebrate feeders
