# Supplementary material for: Incised valleys drive distinctive oceanographic processes and biological assemblages within rhodolith beds
Source: PLoS One. 2023 Nov 13;18(11):e0293259. doi: 10.1371/journal.pone.0293259 (PMC10642839; doi:10.1371/journal.pone.0293259)
Supplement: S2 Table — (DOCX) [file pone.0293259.s004.docx]

S2 Table. Full results of the Permutational Analysis of Variance (PERMANOVA) contrasting reef fish biomass and abundance in the incised valley and adjacent rhodolith bed. **Legends**: Ha= habitat; Si= site

| Reef fish biomass | | |  |  |  |
| --- | --- | --- | --- | --- | --- |
| Source | df | SS | MS | Pseudo-F | P (MC) |
| Ha | 1 | 19659 | 19659 | 8,5198 | 0,0023 |
| Si(Ha) | 2 | 4628,8 | 2314,4 | 1,5716 | 0,1345 |
|  |  |  |  |  |  |
| Reef fish abundance | | |  |  |  |
| Source | df | SS | MS | Pseudo-F | P (MC) |
| Ha | 1 | 19659 | 19659 | 0,341 | 0,0025 |
| Si(Ha) | 2 | 4628,8 | 2314,4 | 1,5716 | 0,1436 |
