# Supplementary material for: Incised valleys drive distinctive oceanographic processes and biological assemblages within rhodolith beds
Source: PLoS One. 2023 Nov 13;18(11):e0293259. doi: 10.1371/journal.pone.0293259 (PMC10642839; doi:10.1371/journal.pone.0293259)
Supplement: S1 Data — (ZIP) [file pone.0293259.s006.zip › Castro_et_al_Suppl_Inf_reworked #2.docx]

**Supporting Information**

**
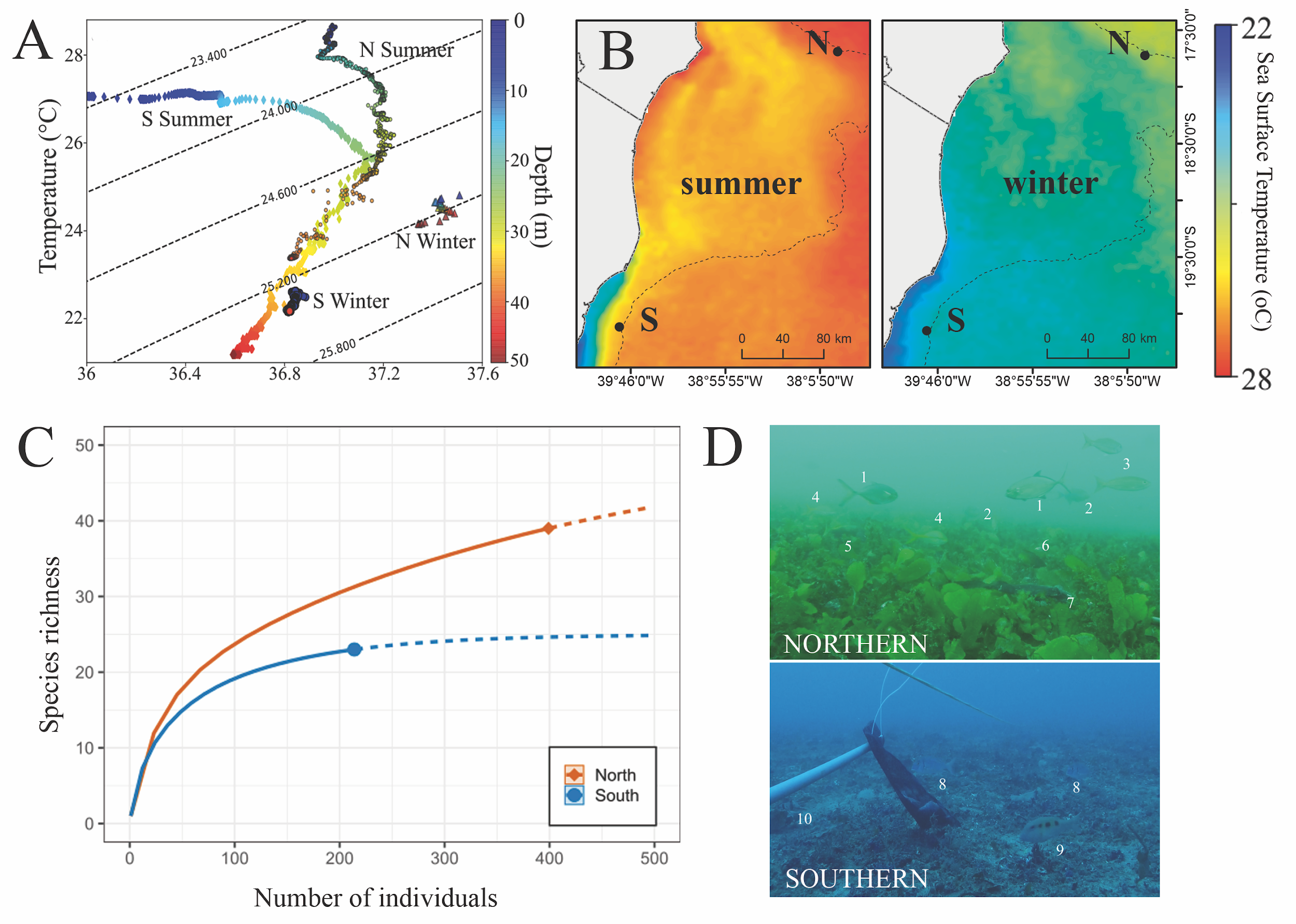
**

S1 Fig. Contrasts between the Northern (N) and Southern (S) Rhodolith Beds (RB) within the tropical-subtropical transition of the Espírito-Santo Abrolhos (ESA) Shelf. A: Temperature-Salinity plots showing stronger summer stratification in the South and a more mixed water column during the winter; B: Summer and winter Sea Surface Temperatures (dashed lines represents the 100 m isobath); C: Rarefaction (solid) and extrapolation curves (dotted) of reef fish richness in the Abrolhos Bank (N) (data from [22]) shown in orange and in the Paleovalley Shelf (S) shown in blue; D: Video frames showing latitudinal contrasts in algal canopies and fish assemblages. Fish identifications: 1 - *Caranx crysos*, 2 - *Balistes vetula*, 3 - *Rhomboplites aurorubens*, 4 - *Ocyurus chrysurus*, 5 - *Haemulon plumierii*, 6 - *Cephalopholis fulva*, 7 - *Gymnothorax moringa*, 8 - *Pagrus pagrus*, 9 - *Pseudupeneus maculatus*, 10 - *Serranus phoebe*.


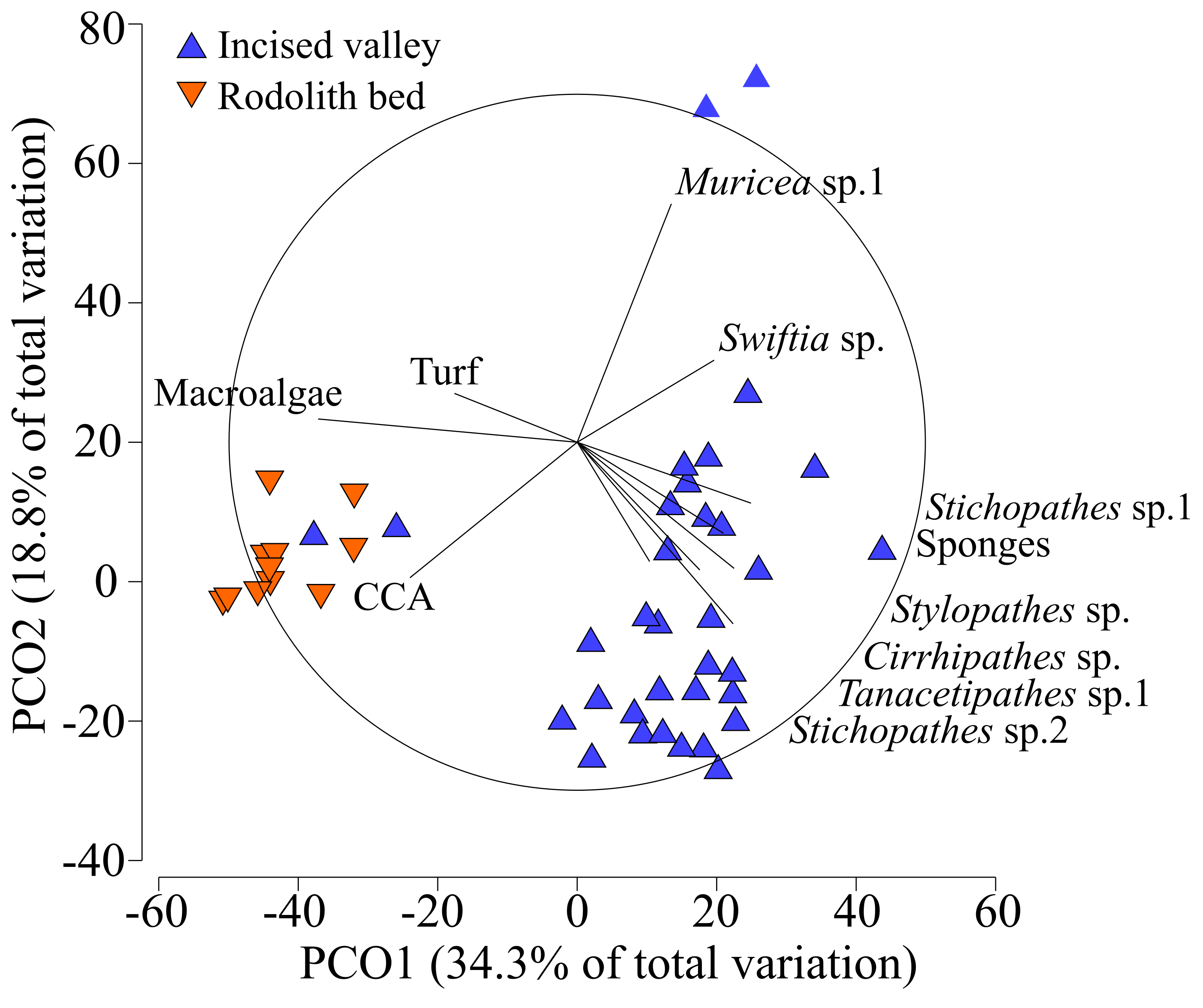


S2 Fig. Principal Coordinate Analysis (PCO) with benthic cover data (%). Sampling strata are color-coded in order to expose benthic habitats contrasts. Only species with the greatest contribution to the ordination are shown.

S1 Table. Checklist of fishes recorded during the survey in the Piraquê-Açu incised valley and adjacent rhodolith bed.

|  |  | | | | | **Rhodolith bed** | | | **Incised valley** | |  |
| --- | --- | --- | --- | --- | --- | --- | --- | --- | --- | --- | --- |
|  | Distribution | Max size (cm) Sampled/ Literature | | Max. Depth (m) | Trophic Guild | | MaxN ± SE | Biomass ± SE | MaxN ± SE | Biomass ± SE | |
|  |  |  |  |  |  |  |  |  |  |  |  |
| **Holocentridae** |  |  |  |  |  | |  |  |  |  | |
| *Holocentrus adscensionis* | WA, OIB, MAR, EA | 26/35 | | 200 | MIN | | NR | | 1.9 ± 1.2 | 0.4 ± 0.3 | |
| *Sargocentron bullisi* |  |  |  |  |  | |  |  |  |  | |
| **Apogonidae** |  |  |  |  |  | |  |  |  |  | |
| *Apogon americanus* | BR, OIB | 7/12 | | 63 | PLA | | NR | | VR | | |
| *Phaeoptyx* *pigmentaria* | WA, OIB, EA | 5/8 | | 70 | PLA | | NR | | VR | | |
| [**Gobiidae**](https://en.wikipedia.org/wiki/Gobiidae) |  |  | |  |  | |  |  |  |  | |
| *Ptereleotris randalli* | BR | 10/12 | | 60 | PLA | | 0.4 ± 0.3 | * | 0.3 ± 0.2 | * | |
| **Pomacentridae** |  |  | |  |  | |  |  |  |  | |
| *Chromis enchrysura* | BR, OIB | 20/13.5 | | 125 | PLA | | NR | | 2.6 ± 1.3 | 0.1 ± 0.1 | |
| *Chromis flavicauda* | BR, OIB | 10/7 | | 120 | PLA | | NR | | 6.3 ± 3.9 | 0.1 ± 0.1 | |
| *Chromis jubauna* | BR, OIB | 14/7 | | 71 | PLA | | NR | | 8.7 ± 5.9 | 0.3 ± 0.2 | |
| *Stegastes pictus* | BR, WA, OIB | 10/7.5 | | 85 | HER | | NR | | 0.8 ± 0.4 | * | |
| **Opistognathidae** |  |  | |  |  | |  |  |  |  | |
| *Opistognathus aurifrons* | BR, OIB | 5/14 | | 65 | PLA | | 0.3 ± 0.3 | * | NR | | |
| **Echeneidae** |  |  | |  |  | |  |  |  |  | |
| *Echeneis naucrates* | CT | 70/110 | | 50 | MCA | | NR | | VR | | |
| **Carangidae** |  |  | |  |  | |  |  |  |  | |
| *Carangoides bartholomaei* | WA, OIB, EA | 32/90 | | 70 | MCA | | 0.1 ± 0.1 | 0.1 ± 0.1 | NR | | |
| *Caranx crysos* | WA, OIB, MAR, EA | 41/70 | | 100 | MCA | | 1.3 ± 0.6 | 0.6 ± 0.2 | 0.1 ± 0.1 | 0.1 ± 0.1 | |
| S1 Table (continued). Checklist of fishes recorded during the survey in the Piraquê-Açu incised valley and adjacent rhodolith bed. | | | | | | | | | | | |
|  |  | | | | | **Rhodolith bed** | | | **Incised valley** | |  |
|  | Distribution | Max size (cm) Sampled/ Literature | | Max. Depth (m) | Trophic Guild | | MaxN ± SE | Biomass ± SE | MaxN ± SE | Biomass ± SE | |
|  |  |  |  |  |  |  |  |  |  |  |  |

| *Caranx latus* | WA, OIB, MAR, EA | 58/100 | 140 | MCA | | NR | | 0.1 ± 0.1 | 0.3 ± 0.3 | |
| --- | --- | --- | --- | --- | --- | --- | --- | --- | --- | --- |
| *Decapterus sp.* | WA, MAR, EA | 36/21 | 90 | MCA | | 5.4 ± 1.9 | 2.4 ± 0.8 | NR | | |
| *Seriola dumerili* | CT | 140/150 | 360 | MCA | | 0.1 ± 0.1 | * | 1.8 ± 0.6 | 33.3 ± 13.5 | |
| **Sphyraenidae** |  |  |  |  | |  |  |  |  | |
| *Sphyraena guachancho* | WA, OIB, EA | 54/200 | 100 | MCA | | 0.2 ± 0.2 | 0.2 ± 0.2 | NR | | |
| **Bothidae** |  |  |  |  | |  |  |  |  | |
| *Bothus* sp. | WA | 25.5/16 | 121 | MIN | | 0.2 ± 0.1 | * | 0.1 ± 0.1 | * | |
| [**Fistulariidae**](https://en.wikipedia.org/wiki/Cornetfish) |  |  |  |  | |  |  |  |  | |
| *Fistularia* sp. | CT | 136/180 | 200 | MCA | | 0.2 ± 0.1 | 0.2 ± 0.2 | 0.2 ± 0.1 | 0.1 ± 0.1 | |
| [**Scombridae**](https://pt.wikipedia.org/wiki/Scombridae) |  |  |  |  | |  |  |  |  | |
| *Scomberomorus* sp. | WA, OIB | 87/184 | 140 | MCA | | NR | | 0.1 ± 0.1 | 0.4 ± 0.4 | |
| **Labridae** |  |  |  |  | |  |  |  |  | |
| *Bodianus pulchellus* | WA, OIB, EA | 25/38 | 120 | MIN | | NR | | 1.1 ± 0.4 | 0.1 ± 0.1 | |
| *Clepticus brasiliensis* | BR, OIB | 25/30 | 62 | PLA | | NR | | 0.1 ± 0.1 | * | |
| *Cryptotomus roseus* | WA, OIB, MAR | 5/13 | 66 | HER | | 0.3 ± 0.3 | * | NR | | |
| *Halichoeres dimidiatus* | BR, OIB | 27/20 | 71 | MIN | | NR | | 0.7 ± 0.2 | 0.1 ± 0.1 | |
| *Halichoeres sazimai* | BR | 17/23 | 190 | MIN | | 0.1 ± 0.1 | * | 1.1 ± 0.8 | * | |
| *Scarus trispinosus* | BR | 59/35.5 | 30 | HER | | NR | | 0.1 ± 0.1 | 0.5 ± 0.5 | |
| *Scarus zelindae* | BR, OIB | 37/33.2 | 55 | HER | | NR | | 0.1 ± 0.1 | 0.1 ± 0.1 | |
| *Sparisoma axillare* | BR, OIB | 33/37 | 45 | HER | | NR | | 0.5 ± 0.3 | 0.2 ± 0.1 | |
| *Sparisoma frondosum* | BR, OIB | 33/34.5 | 45 | HER | | NR | | 0.3 ± 0.2 | 0.3 ± 0.2 | |
| S1 Table (continued). Checklist of fishes recorded during the survey in the Piraquê-Açu incised valley and adjacent rhodolith bed. | | | | | | | | | | |
|  |  | | | | **Rhodolith bed** | | | **Incised valley** | |  |
|  | Distribution | Max size (cm) Sampled/ Literature | Max. Depth (m) | Trophic Guild | | MaxN ± SE | Biomass ± SE | MaxN ± SE | Biomass ± SE | |
|  |  |  |  |  |  |  |  |  |  |  |

| **Mullidae** |  |  |  |  | |  |  |  |  | |
| --- | --- | --- | --- | --- | --- | --- | --- | --- | --- | --- |
| *Pseudupeneus maculatus* | WA, OIB | 27/30 | 90 | MIN | | 2.6 ± 1.4 | 0.5 ± 0.2 | 0.9 ± 0.8 | 0.1 ± 0.1 | |
| *Upeneus parvus* | WA | 22/30 | 112 | MIN | | 2.8 ± 2.8 | 0.2 ± 0.2 | NR | | |
| **Kyphosidae** |  |  |  |  | |  |  |  |  | |
| *Kyphosus* sp. | WA, OIB, MAR, EA | 54/70 | 55 | HER | | NR | | 0.1 ± 0.1 | 0.3 ± 0.3 | |
| **Epinephelidae** |  |  |  |  | |  |  |  |  | |
| *Cephalopholis fulva* | WA, OIB | 34/30 | 218 | MCA | | NR | | 1.9 ± 0.5 | 0.5 ± 0.2 | |
| *Epinephelus morio* | WA | 36/125 | 300 | MCA | | NR | | 0.1 ± 0.1 | 0.1 ± 0.1 | |
| *Mycteroperca acutirostris* | WA | 56/98 | 110 | MCA | | NR | | 0.3 ± 0.1 | 0.7 ± 0.3 | |
| *Mycteroperca bonaci* | WA, OIB | 106/122 | 70 | MCA | | NR | | 0.3 ± 0.2 | 3.5 ± 2.0 | |
| *Paranthias furcifer* | WA, OIB, MAR, EA | 32/40 | 70 | PLA | | NR | | 3.3 ± 1.3 | 0.9 ± 0.4 | |
| **Serranidae** |  |  |  |  | |  |  |  |  | |
| *Diplectrum formosum* | WA, OIB | 27/30 | 80 | MCA | | 1.1 ± 0.4 | 0.1 ± 0.1 | 0.2 ± 0.2 | 0.1 ± 0.1 | |
| *Serranus annularis* | WA, OIB | 8/9 | 70 | MIN | | 0.5 ± 0.3 | * | 0.3 ± 0.2 | * | |
| *Serranus baldwini* | WA, OIB | 6/12 | 80 | MIN | | 0.5 ± 0.3 | * | NR | | |
| *Serranus chionaraia* | WA | 5/5 | 90 | MIN | | 0.6 ± 0.4 | * | NR | | |
| *Serranus phoebe* | WA, OIB | 16/20 | 400 | MIN | | 0.8 ± 0.4 | * | 1.3 ± 0.2 | * | |
| **Chaetodonidae** |  |  |  |  | |  |  |  |  | |
| *Chaetodon sedentarius* | WA, EA | 15/15 | 92 | SIN | | NR | | 1.6 ± 0.5 | 0.1 ± 0.1 | |
| *Chaetodon striatus* | WA, OIB | 13/16 | 65 | SIN | | NR | | 0.6 ± 0.2 | * | |
| S1 Table (continued). Checklist of fishes recorded during the survey in the Piraquê-Açu incised valley and adjacent rhodolith bed. | | | | | | | | | | |
|  |  | | | | **Rhodolith bed** | | | **Incised valley** | |  |
|  | Distribution | Max size (cm) Sampled/ Literature | Max. Depth (m) | Trophic Guild | | MaxN ± SE | Biomass ± SE | MaxN ± SE | Biomass ± SE | |
|  |  |  |  |  |  |  |  |  |  |  |

| *Prognathodes brasiliensis* | BR, OIB | 11/7.5 | 65 | SIN | NR | | 0.4 ± 0.2 | * |
| --- | --- | --- | --- | --- | --- | --- | --- | --- |
| **Pomacanthidae** |  |  |  |  |  |  |  |  |
| *Centropyge aurantonotus* | BR, WA, OIB, EA | 6.5/7.5 | 200 | HER | NR | | 0.2 ± 0.2 | * |
| *Holacanthus ciliaris* | WA, OIB | 30/45 | 120 | SIN | NR | | 0.2 ± 0.1 | 0.1 ± 0.1 |
| *Holacanthus tricolor* | WA, OIB | 9.5/35 | 92 | SIN | NR | | 0.2 ± 0.1 | * |
| *Pomacanthus arcuatus* | WA, OIB | 46/60 | 30 | SIN | NR | | 0.4 ± 0.2 | 1.0 ± 0.5 |
| *Pomacanthus paru* | WA, OIB, MAR | 40/41 | 100 | SIN | NR | | 0.1 ± 0.1 | 0.2 ± 0.2 |
| **Malacanthidae** |  |  |  |  |  |  |  |  |
| *Malacanthus plumieri* | WA, OIB, MAR | 43/70 | 153 | MCA | NR | | 1.5 ± 0.3 | 0.5 ± 0.1 |
| **Haemulidae** |  |  |  |  |  |  |  |  |
| *Anisotremus surinamensis* | WA, OIB | 52/60 | 60 | MIN | NR | | 0.2 ± 0.1 | 0.4 ± 0.3 |
| **Lutjanidae** |  |  |  |  |  |  |  |  |
| *Lutjanus analis* | WA | 73/94 | 95 | MCA | NR | | 0.3 ± 0.2 | 1.6 ± 1.0 |
| *Lutjanus jocu* | WA, OIB, MAR | 45/128 | 70 | MCA | NR | | 0.2 ± 0.2 | 0.3 ± 0.3 |
| *Ocyurus chrysurus* | WA, OIB, EA | 47/86 | 180 | MCA | NR | | 0.1 ± 0.1 | 0.1 ± 0.1 |
| [**Ephippidae**](https://pt.wikipedia.org/wiki/Ephippidae) |  |  |  |  |  |  |  |  |
| *Chaetodipterus faber* | WA | 44/91 | 35 | MIN | NR | | 0.5 ± 0.5 | 1.3 ± 1.3 |
| **Sciaenidae** |  |  |  |  |  |  |  |  |
| *Pareques acuminatus* | WA | 17/23 | 54 | MIN | NR | | VR | |

| S1 Table (continued). Checklist of fishes recorded during the survey in the Piraquê-Açu incised valley and adjacent rhodolith bed. | | | | | | | | | | |
| --- | --- | --- | --- | --- | --- | --- | --- | --- | --- | --- |
|  |  | | | | **Rhodolith bed** | | | **Incised valley** | |  |
|  | Distribution | Max size (cm) Sampled/ Literature | Max. Depth (m) | Trophic Guild | | MaxN ± SE | Biomass ± SE | MaxN ± SE | Biomass ± SE | |
|  |  |  |  |  |  |  |  |  |  |  |
| **Acanthuridae** |  |  |  |  | |  |  |  |  | |
| *Acanthurus bahianus* | BR, WA, OIB, MAR | 24/30 | 71 | HER | | NR | | 0.4 ± 0.3 | 0.1 ± 0.1 | |
| *Acanthurus chirurgus* | WA, OIB, MAR, EA | 36.5/35 | 70 | HER | | NR | | 0.8 ± 0.3 | 0.2 ± 0.1 | |
| *Acanthurus coeruleus* | WA, OIB, MAR | 35/36 | 71 | HER | | NR | | 0.4 ± 0.2 | 0.3 ± 0.2 | |
| **Sparidae** |  |  |  |  | |  |  |  |  | |
| *Calamus* sp. | WA | 35/38 | 86 | MIN | | 0.7 ± 0.2 | 0.2 ± 0.1 | 0.3 ± 0.2 | 0.3 ± 0.1 | |
| *Pagrus pagrus* | WA, EA, OIB | 29/91 | 250 | MIN | | 2.5 ± 0.8 | 0.5 ± 0.2 | NR | | |
| [**Ogcocephalidae**](https://pt.wikipedia.org/wiki/Ogcocephalidae) |  |  |  |  | |  |  |  |  | |
| *Ogcocephalus vespertilio* | WA | 18/30 | 200 | MIN | | NR | | 0.1 ± 0.1 | * | |
| **Ostraciidae** |  |  |  |  | |  |  |  |  | |
| *Acanthostracion sp.* | WA, OIB, EA | 47/55 | 80 | OMN | | NR | | 0.1 ± 0.1 | 0.1 ± 0.1 | |
| **Balistidae** |  |  |  |  | |  |  |  |  | |
| *Balistes capriscus* | WA, OIB, EA | 43/35 | 100 | MIN | | 0.4 ± 0.2 | 0.5 ± 0.2 | NR | | |
| *Balistes vetula* | WA, OIB, MAR, EA | 53.5/50 | 111 | MIN | | NR | | 0.1 ± 0.1 | 0.3 ± 0.3 | |
| **Monacanthidae** |  |  |  |  | |  |  |  |  | |
| *Stephanolepis hispidus* | WA, OIB, EA | 14/27.5 | 293 | OMN | | 0.2 ± 0.1 | * | NR | | |
| **Tetraodontidae** |  |  |  |  | |  |  |  |  | |
| *Lagocephalus laevigatus* | WA, EA | 32/100 | 180 | MCA | | 0.1 ± 0.1 | 0.1 ± 0.1 | NR | | |
| *Sphoeroides camila* | SWA, OIB | 10/30 | 70 | MIN | | NR | | 0.6 ± 0.2 | * | |

**NR** – Not recorded; **VR** – Visual Record; * - < 0.05

Distribution: **WA** - Western Atlantic, **MAR** - Mid Atlantic Ridge, **OIB** - Oceanic Islands of Brazil, **BR** - Brazilian Coast Endemics**, SA** - Southwestern Atlantic, **EA** – Eastern Atlantic; **CT** - Circumtropical

Trophic Guild: **HER –** Herbivores, **MCA –** Macro carnivores, **MIN** – Mobile invertebrate feeders, **OMN** – Omnivore, **PLA** – Planktivore, **SIN** – Sessile invertebrate feeders

S2 Table. Full results of the Permutational Analysis of Variance (PERMANOVA) contrasting reef fish biomass and abundance in the incised valley and adjacent rhodolith bed. **Legends**: Ha= habitat; Si= site

| Reef fish biomass | | |  |  |  |
| --- | --- | --- | --- | --- | --- |
| Source | df | SS | MS | Pseudo-F | P (MC) |
| Ha | 1 | 19659 | 19659 | 8,5198 | 0,0023 |
| Si(Ha) | 2 | 4628,8 | 2314,4 | 1,5716 | 0,1345 |
|  |  |  |  |  |  |
| Reef fish abundance | | |  |  |  |
| Source | df | SS | MS | Pseudo-F | P (MC) |
| Ha | 1 | 19659 | 19659 | 0,341 | 0,0025 |
| Si(Ha) | 2 | 4628,8 | 2314,4 | 1,5716 | 0,1436 |

S1 Video. Supplementary video file.

S1 Data. Raw data.
